# Longitudinal association between cardiovascular risk factors and depression in young people: a systematic review and meta-analysis of cohort studies

**DOI:** 10.1017/S0033291721002488

**Published:** 2023-02

**Authors:** Anna B. Chaplin, Natasha F. Daniels, Diana Ples, Rebecca Z. Anderson, Amy Gregory-Jones, Peter B. Jones, Golam M. Khandaker

**Affiliations:** 1 Department of Psychiatry, University of Cambridge, Cambridge, UK; 2 School of Clinical Medicine, University of Cambridge, Cambridge, UK; 3 Royal Liverpool University Hospital, Liverpool, UK; 4 Liverpool NHS Foundation Trust, Liverpool, UK; 5 Cambridgeshire and Peterborough NHS Foundation Trust, Cambridge, UK; 6 MRC Integrative Epidemiology Unit, Population Health Science, University of Bristol, Bristol, UK; 7 Centre for Academic Mental Health, University of Bristol, Bristol, UK; 8 Avon and Wiltshire Mental Health Partnership NHS Trust, Bristol, UK

**Keywords:** Body mass index, cardiovascular risk factor, depression, depressive symptoms, meta-analysis, smoking

## Abstract

**Background:**

Depression is a common and serious mental illness that begins early in life. An association between cardiovascular disease (CVD) and subsequent depression is clear in adults. We examined associations between individual CVD risk factors and depression in young people.

**Methods:**

We searched MEDLINE, EMBASE, and PsycINFO databases from inception to 1 January 2020. We extracted data from cohort studies assessing the longitudinal association between CVD risk factors [body mass index (BMI), smoking, systolic blood pressure (SBP), total cholesterol, high-density lipoprotein] and depression, measured using a validated tool in individuals with mean age of 24 years or younger. Random effect meta-analysis was used to combine effect estimates from individual studies, including odds ratio (OR) for depression and standardised mean difference for depressive symptoms.

**Results:**

Based on meta-analysis of seven studies, comprising 15 753 participants, high BMI was associated with subsequent depression [pooled OR 1.61; 95% confidence interval (CI) 1.21–2.14; *I*^2^ = 31%]. Based on meta-analysis of eight studies, comprising 30 539 participants, smoking was associated with subsequent depression (pooled OR 1.73; 95% CI 1.36–2.20; *I*^2^ = 74%). Low, but not high, SBP was associated with an increased risk of depression (pooled OR 3.32; 95% CI 1.68–6.55; *I*^2^ = 0%), although this was based on a small pooled high-risk sample of 893 participants. Generalisability may be limited as most studies were based in North America or Europe.

**Conclusions:**

Targeting childhood/adolescent smoking and obesity may be important for the prevention of both CVD and depression across the lifespan. Further research on other CVD risk factors including blood pressure and cholesterol in young people is required.

## Introduction

Depression is a common and serious mental illness with a lifetime risk of 10–20% (Hasin et al., 2018). The majority of depression cases are established by age 24 (Kessler et al., 2010) and this condition is the leading cause of disability among children and young people (Polanczyk, Salum, Sugaya, Caye, & Rohde, 2015). Following an initial depressive episode, the risk of recurrence is 60% (Holtzheimer & Mayberg, 2011). Therefore, early-onset depression is associated with a longer period of risk for relapse as well as poor long-term outcomes (Holtzheimer & Mayberg, 2011; Kessler, 2012). A better understanding of the aetiology of depression in young people is required to develop effective strategies for prevention and treatment (Niarchou, Zammit, & Lewis, 2015).

Cardiovascular disease (CVD) is a leading cause of health-related disability worldwide (Roth et al., 2018). There is evidence for bidirectional associations between CVD and depression in adults (Hiles et al., 2015; Inouye et al., 2018; Khandaker et al., 2019; Smolderen et al., 2017). A substantial body of literature suggests that depression is a key risk factor for CVD in adults and that it may predict poor outcomes following a cardiac event (Barefoot & Schroll, 1996; Hiles et al., 2015; Inouye et al., 2018; Khandaker et al., 2019; Lippi, Montagnana, Favaloro, & Franchini, 2009; Van der Kooy et al., 2007). CVD is also associated with subsequent depression in adults (Choi, Kim, Marti, & Chen, 2014; Hare, Toukhsati, Johansson, & Jaarsma, 2014; Kendler, Gardner, Fiske, & Gatz, 2009; Lippi et al., 2009). However, studies of CVD risk and subsequent depression in young people are relatively less common. A clearer understanding of the association between CVD risk factors and depression in young people is required. Early detection and management of CVD risk factors may reduce risks for both CVD and depression subsequently during the life-course.

The World Health Organization defines young people as individuals aged 24 years or younger (WHO Study Group of Young People, 1986). Existing studies of CVD risk and depression in young people have often focused on individual risk factors such as body mass index (BMI) or smoking. In the past decade, a number of systematic reviews have highlighted an association between obesity in young people and depression across the lifespan (Hoare, Skouteris, Fuller-Tyszkiewicz, Millar, & Allender, 2014; Mannan, Mamun, Doi, & Clavarino, 2016; Mühlig, Antel, Föcker, & Hebebrand, 2016; Sutaria, Devakumar, Yasuda, Das, & Saxena, 2019). However, none of these studies specifically examined depression risk in young people. A recent systematic review of individuals aged 14–35 reported that childhood obesity is associated with approximately 50% increased risk of depression (Sutaria et al., 2019). Another review reported that smoking in early life is associated with 73% increased risk of depression in young people (Chaiton, Cohen, O'Loughlin, & Rehm, 2009). According to the Framingham study, other established CVD risk factors for adults include systolic blood pressure (SBP), total cholesterol, and high-density lipoprotein (HDL), in addition to smoking and BMI (Wilson et al., 1998; Wilson, Castelli, & Kannel, 1987). These CVD risk factors are all potentially modifiable and may be important in the aetiology and prevention of depression. CVD risk factors are increasingly being examined in young people; thus, a systematic review is required to summarise these findings.

We conducted a systematic review and meta-analysis of existing studies to quantify the longitudinal association of five key CVD risk factors (BMI, smoking, SBP, total cholesterol, and HDL) and depression in young people. These CVD risk factors were chosen for a number of reasons: (i) they are part of the Framingham Cardiovascular Risk Score for adults; (ii) they are potentially modifiable; and (iii) they remain relevant in the context of young people. Our outcome was depression (binary or continuous) assessed using a validated tool. We also performed a number of sensitivity analyses, for example by excluding studies that only looked at one gender or excluding studies based on quality assessment.

## Methods

### Search strategy and study selection

This study has been performed according to the Preferred Reporting Items for Systematic Reviews and Meta-Analyses (PRISMA) guidelines. Details of the protocol were prospectively registered on PROSPERO (see https://www.crd.york.ac.uk/PROSPERO/display_record.php?ID=CRD42020172460).

MEDLINE, EMBASE, and PsycINFO databases were searched to identify all relevant studies of the association between CVD risk factors and depression from database inception to 1 January 2020. The following keywords were used: ‘(cohort OR longitudinal OR prospective OR retrospective OR follow up stud*) AND depress* AND (adolescen* OR young person OR young people OR child* OR infant OR early adult OR youth* OR teen*) AND ((*cardiovascular AND risk*) OR total cholesterol OR high density lipoprotein OR hdl OR smok* OR bmi OR body mass index OR adiposity OR waist circumference OR body fat distribution OR skinfold thickness OR lipid accumulation product OR systolic blood pressure OR systolic bp OR sbp)’. See online Supplementary materials for the full search strategy. No language restriction was applied. The electronic search was complemented by hand-searching the reference lists of included studies. All titles and abstracts were examined to retrieve potentially relevant studies. ABC, NFD, AGJ, DP, and RZA applied inclusion/exclusion criteria and selected the final studies for this review.

### Selection criteria

We included studies that: (i) had a longitudinal population-based cohort design (prospective or retrospective); (ii) included participants with a mean age of 24 years old or younger at follow-up; (iii) had at least one of the five CVD risk factors (BMI, smoking, SBP, total cholesterol, HDL) as the exposure at baseline; (iv) used a validated tool to measure depression (binary outcome or symptom score) at follow-up; and (v) reported effect estimate(s) for the association between CVD risk and subsequent depression. Studies were excluded if they did not have an unexposed group for a particular risk factor (e.g. experimental smoking used as the comparison group rather than no smoking), had depression as the exposure and the CVD risk factor as the outcome, or measured depression comorbid with another mental illness such as bipolar disorder or anxiety.

### Data extraction

Data extraction was performed independently by ABC, NFD and DP, and disagreements were resolved by consensus. For each included study, we extracted the following data: (i) details of the cohort (country, name/setting, design, sample size, and follow-up length); (ii) assessment of exposure and outcome; (iii) age and sex of the included participants; and (iv) results of analysis (number of participants exposed at baseline, number of participants with depression at follow-up, adjusted/unadjusted effect estimates). When studies reported various methods for assessing the exposure or repeated measures of the exposure, we used the most comprehensive measure. For example, one study measured BMI eight times from birth to age 12 years (Wang, Leung, & Schooling, 2014). We chose the age 7 measure as the earliest age where BMI may be an appropriate measure of central adiposity to maximise the length of follow-up. In cases where there was more than one published report from the same population, we included the study with the larger sample size (Bares, 2014; Duncan & Rees, 2005; Goodman & Capitman, 2000; Munafò, Hitsman, Rende, Metcalfe, & Niaura, 2008). Some studies reported results where depression at baseline was adjusted for as well as analysis where baseline depression cases were removed. In such cases, we only included results where baseline depression was excluded to minimise reverse causality.

### Data synthesis and meta-analysis

We performed separate meta-analyses for BMI, smoking, and SBP. Results from studies were pooled using the inverse variance method meaning that studies with larger sample sizes were given greater weight (Higgins et al., 2021; Schwarzer, 2007). Results for other CVD risk factors were summarised using a narrative review. Meta-analyses were performed separately for studies that reported beta estimates (continuous depressive symptoms outcome) or odds ratio (OR) (binary depression outcome). We used random-effect meta-analysis, which is appropriate where there is heterogeneity between the studies. Heterogeneity between studies was examined using the *I*^2^ statistic.

We assessed publication bias by visual inspection of funnel plots, and by Egger's regression test for funnel plot asymmetry (mixed-effects meta-regression model). We considered a *p* value of <0.05 to indicate the existence of publication bias.

We assessed study quality using the Newcastle-Ottawa Scale for cohort studies (Stang, 2010). We repeated analyses with only good/fair quality studies. We also repeated analyses after excluding studies with only female or male participants. Meta-analyses were carried out using the *meta* package version 4.11 in R version 3.6.1 (Schwarzer, 2007).

## Results

**Fig. 1.**
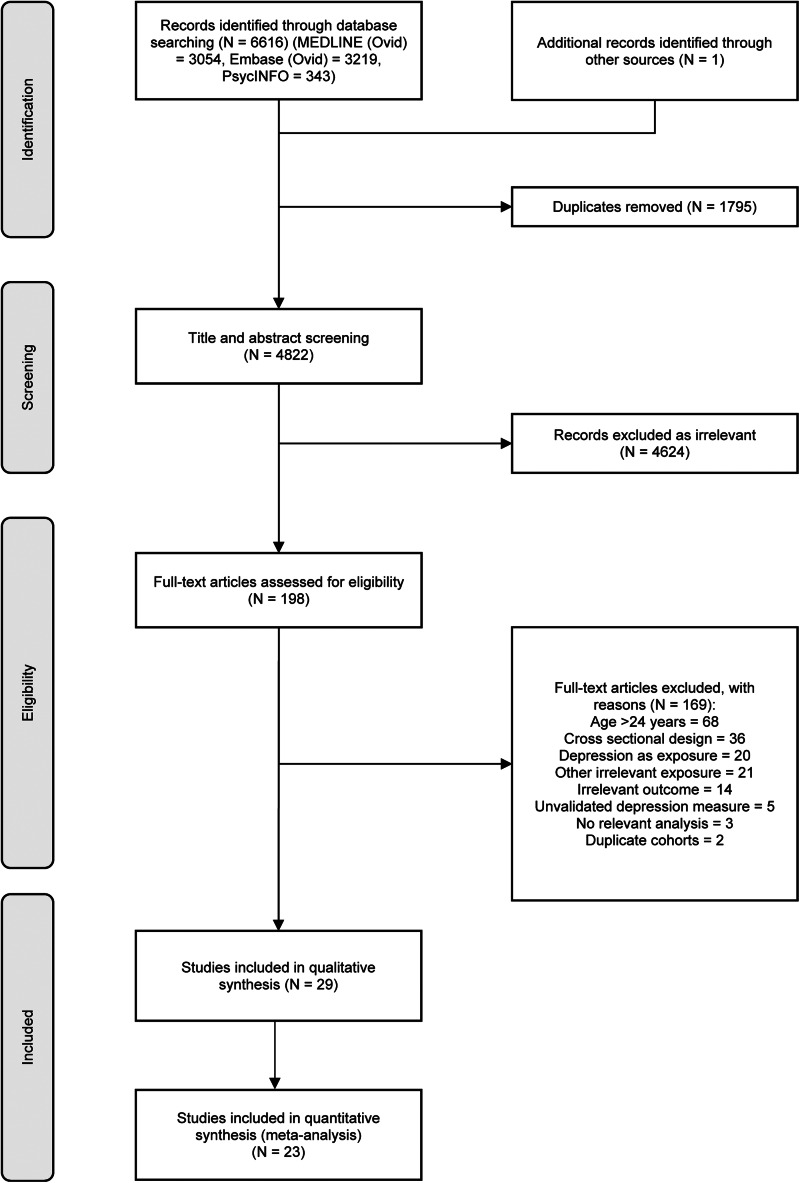
PRISMA flow diagram for study selection.

Electronic search identified 6616 studies. After removing duplicates, 4821 studies remained. After title and abstract screening, 197 (4.1%) potentially eligible studies were identified, of which 29 met the inclusion criteria and were included in the review (Albers & Biener, 2002; Bares, 2014; Beal, Negriff, Dorn, Pabst, & Schulenberg, 2014; Boutelle, Hannan, Fulkerson, Crow, & Stice, 2010; Chaiton, Cohen, Rehm, Abdulle, & O'Loughlin, 2015; Chang et al., 2017; Choi, Patten, Christian Gillin, Kaplan, & Pierce, 1997; Clark et al., 2007; Duncan & Rees, 2005; Eitle & Eitle, 2018; Frisco, Houle, & Lippert, 2013; Gage et al., 2015; Gomes et al., 2019; Goodman & Whitaker, 2002; Hammerton, Harold, Thapar, & Thapar, 2013; Hammerton, Thapar, & Thapar, 2014; Marmorstein, Iacono, & Legrand, 2014; Monshouwer et al., 2012; Perry et al., 2020; Piumatti, 2018; Pryor et al., 2016; Raffetti, Donato, Forsell, & Galanti, 2019; Ranjit et al., 2019*a*, *b*; Rhew et al., 2008; Roberts & Duong, 2013; Rubio, Kraemer, Farrell, & Day, 2008; Wang et al., 2014; Zhang, Woud, Becker, & Margraf, 2018). See Fig. 1 for study selection and Table 1 for characteristics of included studies.

**Table 1.** Characteristics of studies included in systematic review

Study	Country	Female, No. (%)	Baseline age range/mean (years)	Minimum follow-up (years)	Assessment of exposure	Exposed at baseline (%) or mean (s.d.)	Assessment of depression	Depression at follow-up (%)	Mean (s.d.) depressive symptom score at follow-up
*BMI*									
Boutelle et al. (2010)	USA	495 (100)^a^	13.5	1	Obese *v*. healthy weight	10.7%	Structured interview (K-SADS)	1.2–3.5	1.3–1.4 (0.4)
Chang et al. (2017)	Taiwan	969 (51.2)	14.7	3	Obese *v*. healthy weight	3.5% (F); 3.4% (M)	Self-report (CES-DC)	–	1.8 (0.5) (F); 1.5 (0.4) (M)
Clark et al. (2007)	UK	776 (51.3)	11–14	2	Obese *v*. healthy weight	19.6%	Self-report (SMFQ)	15.1	–
Eitle and Eitle (2018)	USA	308 (47.0) (AI); 3403 (50.0) (W)	12–18	1	Obese *v*. not obese	19.0% (AI); 11.0% (W)	Self-report (CES-D)	–	12.4 (0.6) (AI); 10.1 (0.2) (W)
Frisco et al. (2013)	USA	5243 (100)	15.7	4	Consistently obese *v*. healthy weight	9.8%	Self-report (CES-D)	7.0	–
Gomes et al. (2019)	Brazil	NM	18	4	Obese *v*. not obese	6.7%	Structured interview (MINI-5)	2.9	–
Goodman and Whitaker (2002)	USA	4718 (48.6)	<20	1	Obese *v*. not obese	9.7%	Self-report (CES-D)	8.9	–
Hammerton et al. (2014)^b^	UK	EPAD: 165 (57.1);ALSPAC: 2533 (52.1)	EPAD: 9–17; ALSPAC: 11–14	EPAD: 1.4; ALSPAC: 2	Standardised score	EPAD: 17.4%; ALSPAC: 23.7%	EPAD: Semi-structured interview (CAPA); ALSPAC: Structured interview (DAWBA)	EPAD: 8.3; ALSPAC: 1.6	EPAD: 2.2 (NM) (F); 1.6 (NM) (M);ALSPAC: NM
Marmorstein et al. (2014)	USA	NM	11–14	6	Obese *v*. not obese	14.7% (F); 10.7% (M)	Structured interview (SCID)	12.3 (F); 7.6 (M)	–
Monshouwer et al. (2012)	Netherlands	820 (53.3)	10–12	4	Obese *v*. healthy weight	14.1%	Structured interview (WHO CIDI-3)	5.6	–
Perry et al. (2020)^b^	UK	1655 (51.6)	9	9	Raw score	17.5 (2.5)	Self-report (CIS-R)	7.0	3.1 (NM)
Piumatti (2018)^b^	Italy	178 (78.1)	21.4	1	Raw score	21.1 (2.7)	Self-report (PHQ-2)	–	1.1 (0.6)
Pryor et al. (2016)	Canada	661 (54.1)	6–12	1	Early-onset *v*. never overweight	11.0%	Self-report (Kovacs CDI)	–	NM
Rhew et al. (2008)	USA	206 (46.2)	12	1	Overweight *v*. healthy weight	22.4% (F); 30.3% (M)	Structured interview (MFQ)	–	NM
Roberts and Duong (2013)	USA	2040 (48.9) ^a^	11–17	1	Obese *v*. healthy weight	19.7%	Structured interview (DISC IV-Y)	1.7	–
Wang et al. (2014)^b^	Hong Kong	2793 (48.2)	7	7	Standardised score	−0.03 (1.1) (F); 0.3 (1.3) (M)	Self-report (PHQ-9)	–	4.0 (NM)
Zhang et al. (2018)	Germany	1196 (100)	21	1.4	Overweight *v*. healthy weight	7.2%	Structured interview (Diagnostic Interview for Mental Disorders Research Version)	6.5	–
*Smoking*									
Albers and Biener (2002)	USA	259 (49.6) ^a^	12–15	4	Ever *v*. never smoker	33.9%	Self-report (Kandel & Davies)	13.2	–
Bares (2014)	USA	2486 (53.0) ^a^	16.6	5.5	Unit increase in cigarette use	31.3%	Self-report (CES-D)	–	4.9 (3.9)
Beal et al. (2014)	USA	262 (100)	11–20	2	Ever *v*. never smoker	2.9 (3.1)	Self-report (CDI/BDI)	–	46.3 (10.8)
Chaiton et al. (2015)^b^	Canada	416 (49.7)	12–13	5	Smoking initiation *v*. no initiation	47.4%	Self-report (Mellinger scale)	23.8	–
Choi et al. (1997)	USA	3215 (46.8)	12–18	4	Established *v*. never smoker	17.3%	Self-report (Kandel & Davies)	11.5	–
Clark et al. (2007)	UK	776 (51.3)	11–14	2	Tried/regular *v*. never smoker	35.2%	Self-report (SMFQ)	15.1	–
Duncan and Rees (2005)	USA	6748 (51.6)	11–21	1	Smoker *v*. non-smoker	25.8% (F); 25.3% (M)	Self-report (CES-D)	27.0 (F); 21.4 (M)	13.0 (8.7) (F);10.8 (7.5) (M)
Gage et al. (2015)	UK	NM	16	2	Unit increase in cigarette use	9.8%	Self-report (CIS-R)	7.2	–
Raffetti et al. (2019)	Sweden	1636 (51.2)	13–14	1	Current *v*. non-smoker	2.0%	Self-report (CES-DC)	8.3	15.7 (NM)
Ranjit et al. (2019*a*)^b^	Finland	2358 (50.3) ^a^	14	3	Regular *v*. never smoker	9.1%	Self-report (GBI)	5.1 (4.9)	–
Ranjit et al. (2019*b*)^b^	Finland	2174 (51.8) ^a^	17.5	5	Ever *v*. never smoker	25.3%	Self-report (GBI)	4.5 (4.7)	–
Rubio et al. (2008)	USA	278 (100)	23	0.25	Smoker *v*. non-smoker	72.0%	Self-report (CES-D)	85.0	–
Piumatti (2018)	Italy	178 (78.1)	21.4	1	Daily *v*. non-smokers	13.6%	Self-report (PHQ-2)	–	1.1 (0.6)
Zhang et al. (2018)	Germany	1196 (100)	21	1.4	Current *v*. non-smoker	23.6%	Structured interview (Diagnostic Interview for Mental Disorders Research Version)	6.5	–
*Systolic blood pressure*									
Hammerton et al. (2013)	UK	EPAD: 164 (58.4); ALSPAC: 2516 (52.1)	EPAD: 9–17; ALSPAC: 11–14	EPAD: 2.5; ALSPAC: 3	Standardised score	EPAD: 117.3 (13.2); ALSPAC: 111.0 (9.5)	EPAD: Semi-structured interview (CAPA); ALSPAC: Structured interview (DAWBA)	EPAD: 8.5; ALSPAC: 1.6	–

s.d., standard deviation; F,  female; M,   male; AI,  American Indian; W,  White; EPAD,  Early Prediction of Adolescent Depression study; ALSPAC,  Avon Longitudinal Study of Parents and Children; NS,  not significant; NM,  not mentioned; K-SADS,  Kiddie Schedule for Affective Disorders and Schizophrenia; SMFQ,  Short Mood and Feelings Questionnaire; MINI-5,  Mini International Neuropsychiatric Interview Version Five; DSM,  Diagnostic and Statistical Manual of Mental Disorders; CES-D,  Centre for Epidemiological Studies Depression; CAPA,  Child and Adolescent Psychiatric Assessment (Child Version); DAWBA,  Development and Wellbeing Assessment (Child Version); SCID,  Structured Clinical Interview for DSM-III-R; CIS-R,  Clinical Interview Schedule Revised; ICD-10,  International Statistical Classification of Diseases and Related Health Problems Tenth Revision; DISC IV-Y,  Diagnostic Interview Schedule for Children for direct administration to children or adolescents; WHO CIDI-3,  World Health Organisation Composite International Diagnostic Interview Version Three; CDI,  Children's Depression Inventory; PHQ,  Patient Health Questionnaire; MFQ,  Mood and Feelings Questionnaire; GBI,  General Behaviour Inventory; BDI,  Beck's Depression Inventory.

a Baseline sample only.

b Not included in meta-analysis. The four studies not included in BMI meta-analysis were excluded because they measured BMI as a continuous variable. The three studies not included in smoking meta-analysis were excluded because they did not report effect estimates comparable with the other studies.

All studies were prospective in design, except one which used a retrospective measure of depression at follow-up (Monshouwer et al., 2012). The majority of studies (55.2%) were rated as ‘good’ quality using the Newcastle-Ottawa Scale (online Supplementary Tables S1 and S2). Sex, age, parental education, race/ethnicity, baseline depression, and alcohol use were the most commonly used confounders (online Supplementary Table S3).

Based on data availability, meta-analysis for BMI included 13 studies (Boutelle et al., 2010; Chang et al., 2017; Clark et al., 2007; Eitle & Eitle, 2018; Frisco et al., 2013; Gomes et al., 2019; Goodman & Whitaker, 2002; Marmorstein et al., 2014; Monshouwer et al., 2012; Pryor et al., 2016; Rhew et al., 2008; Roberts & Duong, 2013; Zhang et al., 2018), and that for smoking included 11 studies (Albers & Biener, 2002; Bares, 2014; Beal et al., 2014; Chaiton et al., 2015; Choi et al., 1997; Clark et al., 2007; Duncan & Rees, 2005; Gage et al., 2015; Piumatti, 2018; Raffetti et al., 2019; Ranjit et al., 2019*a*, *b*; Rubio et al., 2008; Zhang et al., 2018) (online Supplementary Table S2). Meta-analysis for SBP included one study comprising two separate samples (Hammerton et al., 2013). We found no studies of total cholesterol or HDL that met our inclusion criteria.

### Meta-analysis of adjusted effect estimates

#### Longitudinal association between high BMI at baseline and risk of depression at follow-up

**Fig. 2.**
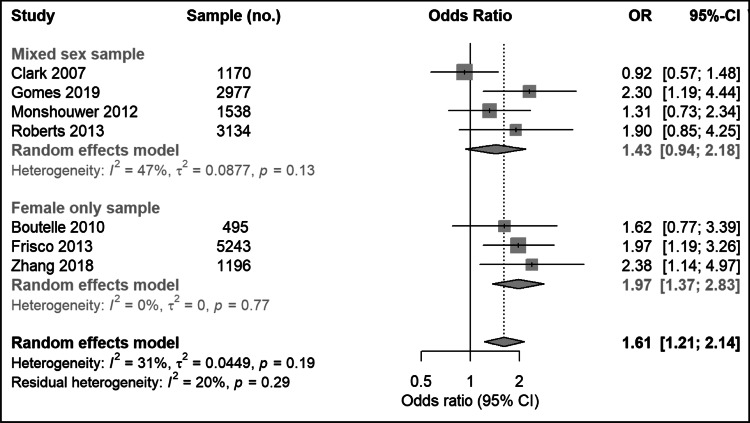
Meta-analysis of longitudinal association between high BMI at baseline and subsequent depression in young people.

Based on seven studies reporting an adjusted OR, comprising a total of 15 753 participants, the pooled OR for depression at follow-up associated with high BMI (>25) at baseline was 1.61 (95% confidence interval (CI)   1.21–2.14) (Fig. 2). There was limited evidence of heterogeneity between studies (*I*^2^ = 31%; 95% CI   0–71%; Cochran's *Q* = 8.7; *p* = 0.19). Separate meta-analysis of unadjusted effect estimates showed lower pooled results (online Supplementary Fig. S1).

#### Longitudinal association between smoking at baseline and risk of depression at follow-up

**Fig. 3.**
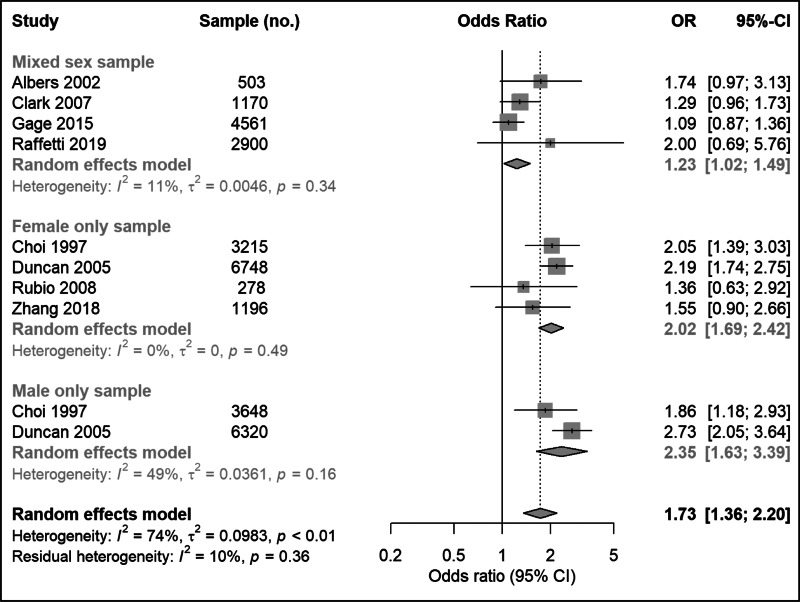
Meta-analysis of longitudinal association between smoking at baseline and subsequent depression in young people.

Based on eight studies reporting an adjusted OR, comprising a total of 30 539 participants, the pooled OR for depression at follow-up associated with smoking at baseline was 1.73 (95% CI   1.36–2.20) (Fig. 3). There was evidence of heterogeneity between studies (*I*^2^ = 74%; 95% CI   52–86%; Cochran's *Q* = 35.3; *p* < 0.01). Separate meta-analysis of unadjusted effect estimates showed higher pooled results (online Supplementary Fig. S2).

#### Longitudinal association between SBP at baseline and risk of depression at follow-up

**Fig. 4.**
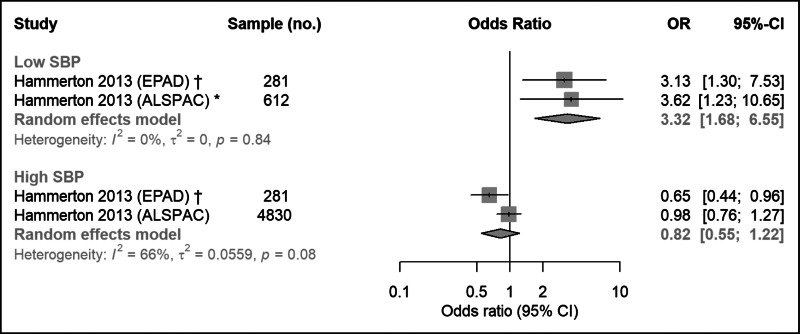
Meta-analysis of longitudinal association between SBP at baseline and subsequent depression in young people. SBP, systolic blood pressure; EPAD, Early Prediction of Adolescent Depression study; ALSPAC, Avon Longitudinal Study of Parents and Children. *Subset of ALSPAC participants with mothers with recurrent depression. ^†^EPAD participants have mother/father with recurrent depression.

One study examined associations of both low and high SBP with depression in two separate samples comprising a total of 5111 participants. Meta-analysis of these studies suggest depression at follow-up is associated with low SBP at baseline (OR   3.32; 95% CI   1.68–6.55), but not with high SBP at baseline (OR   0.82; 95% CI   0.55–1.22) (Fig. 4). There was some evidence of heterogeneity for high SBP (*I*^2^ = 66%; 95% CI   0–92%; Cochran's *Q* = 3.0; *p* = 0.08) and little heterogeneity for low SBP (*I*^2^ = 0%; 95% CI   0–0%; Cochran's *Q* = 0.04; *p* = 0.84).

#### Longitudinal association between high BMI, smoking at baseline and depressive symptoms at follow-up

Based on five studies reporting an adjusted beta estimate, comprising a total of 11 516 participants, the standardised mean difference (SMD) for an increase in depressive symptoms at follow-up associated with high BMI at baseline was 0.05 (95% CI –0.08–0.18) (online Supplementary Fig. S3). There was evidence of heterogeneity between studies (*I*^2^ = 71%; 95% CI  34–88%; Cochran's *Q* = 17.5; *p* < 0.01).

Based on five studies reporting an adjusted beta estimate, comprising a total of 21 490 participants, the SMD for an increase in depressive symptoms at follow-up associated with smoking at baseline was 0.37 (95% CI   0.10–0.64) (online Supplementary Fig. S3). There was evidence of heterogeneity between studies (*I*^2^ = 89%; 95% CI   78–94%; Cochran's *Q* = 44.8; *p* < 0.01).

### Results for sensitivity analysis

After excluding three studies with only female participants (Boutelle et al., 2010; Frisco et al., 2013; Zhang et al., 2018), the adjusted pooled OR for depression at follow-up for high BMI at baseline was 1.43 (95% CI   0.94–2.18) (Fig. 2). There was some heterogeneity between studies (*I*^2^ = 47%; 95% CI   0–83%; Cochran's *Q* = 5.7; *p* = 0.13).

After excluding four studies with only female or male participants (Choi et al., 1997; Duncan & Rees, 2005; Rubio et al., 2008; Zhang et al., 2018), the pooled adjusted OR for depression at follow-up for smoking at baseline was 1.23 (95% CI   1.02–1.49) (Fig. 3). There was little heterogeneity (*I*^2^ = 11%; 95% CI   0–86%; Cochran's *Q* = 3.4; *p* = 0.34).

After excluding one study based on quality assessment (Duncan & Rees, 2005), the pooled adjusted OR for depression at follow-up for smoking at baseline was 1.48 (95% CI   1.21–1.80) (online Supplementary Fig. S4). There was some heterogeneity (*I*^2^ = 38%; 95% CI   0–73%; Cochran's *Q* = 11.3; *p* = 0.12). For BMI, we did not exclude any studies based on quality assessment.

See online Supplementary Figs S5 and S6 for sensitivity analyses where the outcome of interest was depressive symptoms at follow-up.

### Publication bias

Based on Egger's test and funnel plots, evidence for publication bias was not evident for studies reporting the adjusted association between high BMI and depression (Egger's test: *p* = 0.17; online Supplementary Fig. S7), smoking and depression (Egger's test: *p* = 0.80; online Supplementary Fig. S8), or high BMI and depressive symptoms (Egger's test: *p* = 0.35) (online Supplementary Fig. S9). Evidence for publication bias was present for studies reporting the adjusted association between smoking and depressive symptoms (Egger's test: *p* = 0.01) (online Supplementary Fig. S10).

## Discussion

Depression and CVD are associated with each other in mid to late adulthood. Although depression is known to arise commonly in young people, the timing of the association with CVD risk is unclear and common risk factors for the two conditions raise the prospect of joint prevention. To the best of our knowledge, this is the first systematic review to consider the association between various CVD risk factors and subsequent depression in young people. We report four key findings: (i) BMI and smoking are the most well-studied risk factors for depression in this age group; (ii) both BMI and smoking at baseline are longitudinally associated with subsequent depression; (iii) smoking but not BMI is prospectively associated with depressive symptoms; and (iv) currently there is limited data on longitudinal associations of high SBP and cholesterol with subsequent depression in young people, which should be examined in future.

Our results suggest that obesity could be an important risk factor for depression in young people. The pooled OR of 1.61 for the association of high BMI and depression is remarkably similar to previous studies in adults. Previous meta-analyses have reported ORs of 1.51 and 1.70 for the prospective association between childhood high BMI and adult depression (Luppino et al., 2010; Sutaria et al., 2019). Another meta-analysis reported that obese adolescents had an 40% increased risk of experiencing depression as adults (Mannan et al., 2016). A recent Mendelian randomisation study using data from 812 000 adult participants also found that fat mass could be a causal factor for depression (Speed, Jefsen, Børglum, Speed, & Østergaard, 2019). We did not find an association between high BMI and depressive symptoms score, indicating a possibly non-linear association between BMI and depression whereby association is restricted to only those with more severe symptoms.

Our findings also suggest that smoking could be a risk factor for depression in young people. The pooled OR of 1.73 for the association between smoking and depression is consistent with a previous meta-analysis in adults, which reported an OR of 1.62 (Luger, Suls, & Vander Weg, 2014). Similarly, a meta-analysis of nine cross-sectional and longitudinal studies found that adolescents exposed to second-hand smoking had increased odds of depression (Han, Liu, Gong, Ye, & Zhou, 2019). However, current evidence from observational cohort studies and genetic Mendelian randomisation studies reports mixed findings regarding the association between smoking and depression, with some reporting an association (Wootton et al., 2020) and others no association (Bjørngaard et al., 2013; Taylor et al., 2014). Therefore, residual confounding or reverse causality remain viable explanations for the observed association between smoking and depression. Further longitudinal studies and genetic Mendelian randomisation studies are required to investigate this issue.

A number of potential mechanisms may be involved in the association of high BMI and subsequent depression, including low-grade systemic inflammation, hypothalamic–pituitary–adrenal axis (HPA) axis dysregulation, insulin resistance, and psychological distress. Inflammation is evident in around 25% of individuals with depression (Osimo, Baxter, Lewis, Jones, & Khandaker, 2019) and atypical depression is associated with inflammation and metabolic dysregulation (Lamers et al., 2013, 2020). Adipose tissue also contains abundant inflammatory cytokines that are involved in fat metabolism (Heredia, Gómez-Martínez, & Marcos, 2012). Similarly, the role of insulin in regulating adipocyte function contributes to the close link between insulin resistance and obesity (Kahn & Flier, 2000). Furthermore, melancholic depression, higher levels of abdominal fat, HPA axis hyperactivity, and cortisol dysregulation are inter-related (Incollingo Rodriguez et al., 2015; Lamers et al., 2013, 2020). Adverse childhood experiences are also one of the most robust risk factors for depression (Gardner, Thomas, & Erskine, 2019), and are associated with increased risk for obesity and metabolic dysregulation (Farr et al., 2015). Low-grade systemic inflammation may be important to the association between smoking and depression (Berk et al., 2013). Studies are required to investigate the complex mechanisms that may underlie the association between high BMI and depression.

Low SBP, but not high SBP, appeared to be associated with risk for depression in young people. However, our meta-analysis was based on only two cohorts at high risk for depression and further study is required. Low SBP appears to be associated with depression in young people at high-risk of depression but not in the general population (Hammerton et al., 2013). Low SBP has also been associated with depression in cross-sectional and longitudinal studies of middle-aged and elderly adults (Hildrum et al., 2007; Huang, Su, Jiang, & Zhu, 2020). Conversely, higher SBP has been prospectively associated with fewer depressive symptoms in older adults with CVD risk factors (Herrmann-Lingen et al., 2018). In adult populations, the relationship between SBP and depression may be independent of a range of lifestyle factors, age, and sex (Hildrum et al., 2007; Huang et al., 2020).

The reason why low SBP has a potentially causal role in depression remains unclear. Neurons controlling blood pressure could be implicated in the association between SBP and depression. Neuropeptide Y, for example, reduces blood pressure and is involved in stress responses that have been linked to increased risk for depression, such as the HPA axis (Hildrum et al., 2007; Juruena, Bocharova, Agustini, & Young, 2018). Further research is required to understand the relationship between SBP and depression in young people, including potential mechanisms.

We found no studies assessing the association between either total cholesterol or HDL on risk for subsequent depression in young people. A meta-analysis of 30 cross-sectional studies reported that higher total cholesterol was associated with lower levels of depression in adults (Shin, Suls, & Martin, 2008). Evidence from adults indicates both higher and lower HDL to be associated with increased risk for depression in adults. In a meta-analysis of 16 cross-sectional studies, high HDL was related to higher levels of depression, especially in women (Shin et al., 2008). Conversely, a meta-analysis of 11 case-control studies reported that lower HDL levels may be associated with first-episode major depressive disorder in adults (Wei et al., 2020). Given that abnormal HDL, LDL and triglyceride levels are increasingly common in adolescents [Centers for Disease Control and Prevention (CDC), 2010], effort should be made to study potential effects on mental health as well as physical health.

Strengths of this study include the systematic literature search which identified a large number of relevant studies comprising a total of 93 021 participants. We included studies considering the effect of various CVD risk factors on either binary or continuous measures of depression/depressive symptoms. We assessed the studies using the validated Newcastle-Ottawa Scale as well as conducting sensitivity analyses to examine the robustness of our findings. However, this study is not without limitations. First, the majority of studies came from North America and Europe, limiting the generalisability of the results to other parts of the world. The number of studies in each of the meta-analyses was also relatively small, which resulted in wide CIs for the pooled effect estimates, and reduced the statistical power to detect publication bias. There was a considerable amount of heterogeneity between studies, particularly studies of depressive symptoms. Sensitivity analyses revealed that sex explained heterogeneity in some of the meta-analyses. However, stratifying by sex decreased the sample size, and consequently, statistical power to detect an association. In future, studies with larger samples are required. Finally, the possibility of residual confounding by unidentified factors remains high so we are cautious in terms of any conclusion regarding causality. Although studies included in this meta-analysis controlled for various potential confounding effects, other factors may also explain these associations. Further research is needed to examine whether observed associations are likely to be causal. Since randomised controlled trials are neither feasible nor ethical for some of the exposures under investigation (e.g. smoking, obesity), genetic approaches to dealing with residual confounding, such as Mendelian randomisation, would be particularly useful.

In summary, we present evidence for a longitudinal association between CVD risk factors, namely high BMI and smoking, in childhood/adolescence and subsequent depression in young people. These risk factors could be important targets for the prevention of depression and CVD in young people and subsequently during the life course. Further study is needed to understand potential mechanisms for these associations as well as the relationship between other CVD risk factors, notably blood pressure and cholesterol and depression risk in young people.

## Supplementary material

For supplementary material accompanying this paper visit https://doi.org/10.1017/S0033291721002488. click here to view supplementary material
