# Supplementary material for: Longitudinal association between cardiovascular risk factors and depression in young people: a systematic review and meta-analysis of cohort studies
Source: Psychol Med. 2021 Jun 25;53(3):1049–59. doi: 10.1017/S0033291721002488 (PMC9975997; doi:10.1017/S0033291721002488)
Supplement: Supplementary file 1 [file S0033291721002488sup001.docx]

**Supplementary Material**

**Contents**

[1. Search Strategy 25](#_Toc60133577)

[2. Quality Assessment 28](#_Toc60133578)

[3. Characteristics 29](#_Toc60133579)

[4. Confounders 30](#_Toc60133580)

[5. Meta-Analyses of Unadjusted Results 31](#_Toc60133581)

[6. Meta-Analysis of Adjusted Results for Depressive Symptoms 33](#_Toc60133582)

[7. Sensitivity Analysis for Adjusted Results 34](#_Toc60133583)

[8. Publication Bias 37](#_Toc60133584)

[9. References 41](#_Toc60133585)

## **1. Search Strategy**

**MEDLINE search**

1. exp Cohort Studies/
2. (Cohort analy* or longitudinal or prospective or retrospective or ((cohort or follow up) adj (study or studies))).ti,ab.
3. 1 or 2
4. Depression/ or exp Depressive Disorder/
5. depress*.ti,ab.
6. 4 or 5
7. adolescent/ or young adult/ or exp child/ or exp infant/
8. (adolescen* or “young person” or “young people” or child or children or childhood or infant or “early adult*” or youth* or teen*).ti,ab.
9. 7 or 8
10. exp Cardiovascular Diseases/
11. cardiovascular.ti,ab.
12. 10 or 11
13. exp Risk/
14. risk.ti,ab.
15. 13 or 14
16. 12 and 15
17. ((cardiovascular or CVD or heart disease or cardiometabolic or coronary artery disease or CAD or atherosclerosis) adj3 risk).ti,ab.
18. 16 or 17
19. cholesterol/ or cholesterol, dietary/ or lipoproteins/ or exp lipoproteins, hdl/
20. (“total cholesterol” or cholesterol or “high density lipoprotein*” or hdl).ti,ab.
21. 15 or 16
22. exp Smoking/ or Smokers/ or Non-Smokers/ or Ex-Smokers/
23. smok*.ti,ab.
24. 18 or 19
25. body fat distribution/ or adiposity/ or body mass index/ or waist circumference/ or skinfold thickness/ or lipid accumulation product/
26. (BMI or “body mass index” or “body fat distribution” or adiposity or “waist circumference” or WC or “skinfold thickness” or “lipid accumulation product” or LAP).ti,ab.
27. 21 or 22
28. Blood Pressure/
29. (“systolic blood pressure” or “systolic BP” or SBP).ti,ab.
30. 24 or 25
31. 18 or 21 or 24 or 27 or 30
32. 3 and 6 and 9 and 29

**EMBASE search**

1. Longitudinal study/ or Retrospective study/ or Prospective study/ or Cohort analysis/
2. (Cohort analy* or longitudinal or prospective or retrospective or ((cohort or follow up) adj (study or studies))).ti,ab.
3. 1 or 2
4. exp depression/
5. depress*.ti,ab.
6. 4 or 5
7. exp adolescent/ or young adult/ or exp child/
8. (adolescen* or “young person” or “young people” or child or children or childhood or infant or “early adult*” or youth* or teen*).ti,ab.
9. 7 or 8
10. exp cardiovascular risk/ or cardiometabolic risk/
11. ((cardiovascular or CVD or heart disease or cardiometabolic or coronary artery disease or CAD or atherosclerosis) adj3 risk).ti,ab.
12. 10 or 11
13. cholesterol/ or exp high density lipoprotein cholesterol level/ or exp cholesterol blood level/ or total cholesterol level/ or cholesterol level/ or exp high density lipoprotein cholesterol/
14. (“total cholesterol” or cholesterol or “high density lipoprotein*” or hdl).ti,ab.
15. 13 or 14
16. exp smoking/ or exp adolescent smoking/
17. smok*.ti,ab.
18. 16 or 17
19. exp obesity/ or exp body mass/ or exp waist circumference/ or exp skinfold thickness/ or exp lipid accumulation product index/
20. (BMI or “body mass index” or “body fat distribution” or adiposity or “waist circumference” or WC or “skinfold thickness” or “lipid accumulation product” or LAP).ti,ab.
21. 19 or 20
22. exp systolic blood pressure/
23. (“systolic blood pressure” or “systolic BP” or SBP).ti,ab.
24. 22 or 23
25. 12 or 15 or 18 or 21 or 24
26. 3 and 6 and 9 and 25

**PsycINFO search**

1. DE "Longitudinal Studies" OR DE "Prospective Studies" OR DE "Retrospective Studies" OR DE "Followup Studies" OR DE "Cohort Analysis"
2. MA cohort analy* OR MA longitudinal OR MA prospective OR MA retrospective OR MA ((cohort or follow up) n (study or studies))
3. 1 OR 2
4. MM "Major Depression" OR MM "Anaclitic Depression" OR MM "Dysthymic Disorder" OR MM "Endogenous Depression" OR MM "Late Life Depression" OR MM "Postpartum Depression" OR MM "Reactive Depression" OR MM "Recurrent Depression" OR MM "Treatment Resistant Depression"
5. MA depress*
6. 4 OR 5
7. DE "Early Adolescence" OR DE "Emerging Adulthood" OR DE "Postnatal Period" OR DE "Puberty"
8. MA adolescen* OR MA “young person” OR MA “young people” OR MA child OR MA children OR MA childhood OR MA infant OR “early adulthood” OR youth* OR MA teen*
9. 7 OR 8
10. MM "Cardiovascular Disorders" OR MM "Aneurysms" OR MM "Arteriosclerosis" OR MM "Embolisms" OR MM "Heart Disorders" OR MM "Ischemia" OR MM "Thromboses"
11. MA cardiovascular
12. 10 OR 11
13. MM "Risk Factors"
14. MA risk
15. 13 OR 14
16. 12 AND 15
17. (MA cardiovascular OR MA CVD OR MA heart disease OR MA cardiometabolic OR MA coronary artery disease OR MA CAD OR MA atherosclerosis) n3 risk
18. 16 OR 17
19. MM “Cholesterol”
20. MA total cholesterol OR MA cholesterol OR MA high density lipoprotein* OR MA hdl
21. 19 OR 20
22. MM "Tobacco Smoking" OR MM "Electronic Cigarettes" OR MM "Passive Smoking" OR MM "Smokeless Tobacco"
23. MA smok*
24. 22 OR 23
25. MM "Body Mass Index" AND MM "Body Weight" OR MM "Overweight" OR MM "Obesity" OR MM "Weight Gain" OR MM "Body Fat"
26. MA BMI OR MA “body mass index” OR MA “body fat distribution” OR MA adiposity OR MA “waist circumference” OR MA WC OR MA “skinfold thickness” OR MA “lipid accumulation product” OR MA LAP
27. 25 OR 26
28. MM "Systolic Pressure"
29. MA “systolic blood pressure” OR MA “systolic BP” OR MA SBP
30. 28 OR 29
31. 18 OR 21 OR 24 OR 27 OR 30
32. 3 AND 6 AND 9 AND 31

| **S1 Table. Breakdown of Newcastle-Ottawa Scale (NOS) scores for included studies.** | | | | | |
| --- | --- | --- | --- | --- | --- |
| **Study** | **Selection**  **(out of 4)** | **Comparability**  **(out of 2)** | **Outcome**  **(out of 3)** | **Total** | **Rating** |
| Albers 2002 | 4 | 2 | 2 | 8 | Good |
| Bares 2014 | 3 | 2 | 2 | 7 | Good |
| Beal 2014 | 2 | 2 | 3 | 7 | Fair |
| Boutelle 2010 | 3 | 1 | 3 | 7 | Good |
| Chaiton 2015 | 3 | 2 | 2 | 7 | Good |
| Chang 2017 | 2 | 2 | 3 | 7 | Fair |
| Choi 1997 | 3 | 2 | 3 | 8 | Good |
| Clark 2007 | 3 | 2 | 2 | 7 | Good |
| Duncan 2005 | 3 | 2 | 1 | 6 | Poor |
| Eitle 2018 | 2 | 2 | 3 | 7 | Fair |
| Frisco 2013 | 4 | 2 | 3 | 9 | Good |
| Gage 2015 | 3 | 1 | 2 | 6 | Good |
| Gomes 2019 | 4 | 1 | 2 | 7 | Good |
| Goodman 2002 | 2 | 2 | 2 | 6 | Fair |
| Hammerton 2014 * | 3/4 | 0 | 3/2 | 6 | Poor |
| Hammerton 2013 * | 3/4 | 0 | 3/2 | 6 | Poor |
| Marmorstein 2014 | 3 | 0 | 2 | 5 | Poor |
| Monshouwer 2012 | 3 | 1 | 3 | 7 | Good |
| Perry 2020 | 3 | 1 | 2 | 6 | Good |
| Piumatti 2018 | 1 | 2 | 1 | 4 | Poor |
| Pryor 2016 | 3 | 1 | 2 | 6 | Good |
| Raffetti 2019 | 3 | 1 | 2 | 6 | Good |
| Ranjit 2019a | 1 | 2 | 2 | 4 | Poor |
| Ranjit 2019b | 1 | 2 | 1 | 5 | Poor |
| Rhew 2008 | 1 | 1 | 3 | 5 | Poor |
| Roberts 2013 | 4 | 2 | 2 | 8 | Good |
| Rubio 2008 | 2 | 2 | 2 | 6 | Fair |
| Wang 2014 | 3 | 2 | 2 | 7 | Good |
| Zhang 2018 | 3 | 1 | 2 | 6 | Good |
| *Two scores given because there are two cohorts in these papers | | | | | |

## **2. Quality Assessment**

## **3. Characteristics**

| **S2 Table. Summary of characteristics of studies included in the systematic review.** | |
| --- | --- |
| **Characteristic** | **Value** |
| **Cohort type – no. papers (%)**  Prospective  Retrospective | 28 (96.6)  1 (3.4) |
| **Location – no. papers (%)** |  |
| North America | 15 (51.7) |
| Europe | 11 (37.9) |
| Asia | 2 (6.9) |
| South America | 1 (3.4) |
| **Sex – no. papers (%)** |  |
| Female and male | 24 (82.8) |
| Female only | 5 (17.2) |
| **Exposure – no. papers (%) *** |  |
| BMI | 17 (58.6) |
| Smoking | 14 (48.3) |
| Systolic blood pressure | 1 (3.4) |
| Total cholesterol | 0 (0) |
| HDL | 0 (0) |
| **Mean (SD)** |  |
| Follow-up length (years) | 2.9 (2.1) |
| Sample size (N) | 3208 (3004) |
| **Baseline depression – no. papers (%)** |  |
| Baseline cases controlled for/excluded | 22 (75.9) |
| No action | 6 (24.1) |
| **Regression type – no. papers (%) *** |  |
| Logistic | 19 (65.5) |
| Linear | 14 (48.3) |
| **NOS quality rating – no. papers (%)** |  |
| Good | 16 (55.2) |
| Poor | 8 (27.6) |
| Fair | 5 (17.2) |
| * Some papers are counted in more than one category (percentages add up to more than 100%). | |

## **4. Confounders**

| **S3 Table. Number (%) of studies that adjust for various potential confounders in adjusted analysis.** | | |
| --- | --- | --- |
| **Potential confounder** | **No. (%) of studies including confounder** | **Study References** |
| Sex | 20 (71.4) | (Albers & Biener, 2002; Chaiton et al., 2015; Chang et al., 2017; Choi et al., 1997; Clark et al., 2007; Eitle & Eitle, 2018; Gage et al., 2015; Gomes et al., 2019; Goodman & Whitaker, 2002; Marmorstein et al., 2014; Monshouwer et al., 2012; Perry et al., 2020; Piumatti, 2018; Pryor et al., 2016; Raffetti et al., 2019; Ranjit, Buchwald, et al., 2019; Ranjit, Korhonen, et al., 2019; Rhew et al., 2008; Roberts & Duong, 2013; Wang et al., 2014) |
| Age | 15 (53.6) | (Albers & Biener, 2002; Bares, 2014; Beal et al., 2014; Boutelle et al., 2010; Chang et al., 2017; Choi et al., 1997; Clark et al., 2007; Duncan & Rees, 2005; Eitle & Eitle, 2018; Frisco et al., 2013; Goodman & Whitaker, 2002; Piumatti, 2018; Ranjit, Korhonen, et al., 2019; Roberts & Duong, 2013; Rubio et al., 2008; Wang et al., 2014) |
| Parental education | 13 (46.4) | (Albers & Biener, 2002; Chang et al., 2017; Choi et al., 1997; Duncan & Rees, 2005; Eitle & Eitle, 2018; Frisco et al., 2013; Gage et al., 2015; Gomes et al., 2019; Goodman & Whitaker, 2002; Perry et al., 2020; Raffetti et al., 2019; Ranjit, Korhonen, et al., 2019; Rhew et al., 2008) |
| Race/ethnicity | 11 (39.3) | (Albers & Biener, 2002; Beal et al., 2014; Choi et al., 1997; Clark et al., 2007; Duncan & Rees, 2005; Frisco et al., 2013; Gomes et al., 2019; Goodman & Whitaker, 2002; Perry et al., 2020; Rhew et al., 2008; Rubio et al., 2008) |
| Baseline depression | 11 (39.3) | (Albers & Biener, 2002; Beal et al., 2014; Boutelle et al., 2010; Chang et al., 2017; Clark et al., 2007; Goodman & Whitaker, 2002; Pryor et al., 2016; Raffetti et al., 2019; Ranjit, Buchwald, et al., 2019; Ranjit, Korhonen, et al., 2019; Rhew et al., 2008) |
| Alcohol use | 9 (32.1) | (Chaiton et al., 2015; Clark et al., 2007; Gage et al., 2015; Gomes et al., 2019; Piumatti, 2018; Raffetti et al., 2019; Ranjit, Buchwald, et al., 2019; Ranjit, Korhonen, et al., 2019; Zhang et al., 2018) |
| Physical health | 7 (25.0) | (Clark et al., 2007; Duncan & Rees, 2005; Frisco et al., 2013; Perry et al., 2020; Ranjit, Buchwald, et al., 2019; Rhew et al., 2008; Zhang et al., 2018) |
| Family/household structure | 6 (21.4) | (Chang et al., 2017; Duncan & Rees, 2005; Eitle & Eitle, 2018; Frisco et al., 2013; Goodman & Whitaker, 2002; Ranjit, Korhonen, et al., 2019; Rubio et al., 2008) |
| Family/household income | 6 (21.4) | (Bares, 2014; Choi et al., 1997; Frisco et al., 2013; Gomes et al., 2019; Piumatti, 2018; Rhew et al., 2008; Roberts & Duong, 2013) |
| Academic performance | 6 (21.4) | (Choi et al., 1997; Eitle & Eitle, 2018; Piumatti, 2018; Ranjit, Buchwald, et al., 2019; Ranjit, Korhonen, et al., 2019; Rubio et al., 2008) |
| Weight/BMI | 5 (17.9) | (Clark et al., 2007; Gomes et al., 2019; Hammerton et al., 2013; Piumatti, 2018; Zhang et al., 2018) |
| Physical activity | 5 (17.9) | (Choi et al., 1997; Frisco et al., 2013; Perry et al., 2020; Roberts & Duong, 2013; Zhang et al., 2018) |
| Socioeconomic status | 5 (17.9) | (Beal et al., 2014; Clark et al., 2007; Duncan & Rees, 2005; Perry et al., 2020; Wang et al., 2014) |
| Pubertal timing | 4 (14.3) | (Beal et al., 2014; Boutelle et al., 2010; Chang et al., 2017; Pryor et al., 2016) |
| Parental smoking | 4 (14.3) | (Albers & Biener, 2002; Bares, 2014; Beal et al., 2014; Gomes et al., 2019; Ranjit, Korhonen, et al., 2019) |
| Cigarette smoking | 4 (14.3) | (Clark et al., 2007; Gomes et al., 2019; Piumatti, 2018; Zhang et al., 2018) |
| Drug use | 3 (10.7) | (Clark et al., 2007; Gage et al., 2015; Rubio et al., 2008) |
| Parental depression | 3 (10.7) | (Monshouwer et al., 2012; Perry et al., 2020; Wang et al., 2014) |
| Peer victimisation | 3 (10.7) | (Chang et al., 2017; Gage et al., 2015; Pryor et al., 2016) |
| Cannabis use | 2 (7.1) | (Gage et al., 2015; Piumatti, 2018) |
| Family functioning | 2 (7.1) | (Monshouwer et al., 2012; Pryor et al., 2016) |
| Urban dwelling | 2 (7.1) | (Duncan & Rees, 2005; Gage et al., 2015) |
| Parental BMI | 2 (7.1) | (Gomes et al., 2019; Goodman & Whitaker, 2002) |
| Birthplace | 2 (7.1) | (Frisco et al., 2013; Raffetti et al., 2019) |
| Parenthood | 2 (7.1) | (Gomes et al., 2019; Rubio et al., 2008) |
| Diet | 1 (3.6) | (Roberts & Duong, 2013) |
| Maternal age | 1 (3.6) | (Gomes et al., 2019) |
| Availability of social support | 1 (3.6) | (Choi et al., 1997) |
| Family economic stress | 1 (3.6) | (Chang et al., 2017) |
| Other mental health problems | 1 (3.6) | (Gage et al., 2015) |
| Maternal mental health | 1 (3.6) | (Gomes et al., 2019) |
| Family history of depression | 1 (3.6) | (Gage et al., 2015) |
| Alcoholic parent | 1 (3.6) | (Duncan & Rees, 2005) |
| Impulsivity | 1 (3.6) | (Chaiton et al., 2015) |
| Rebelliousness | 1 (3.6) | (Choi et al., 1997) |
| Stressful life events | 1 (3.6) | (Chang et al., 2017) |
| IQ | 1 (3.6) | (Gage et al., 2015) |
| Ever pregnant | 1 (3.6) | (Frisco et al., 2013) |
| Tiredness | 1 (3.6) | (Monshouwer et al., 2012) |
| Sleep problems | 1 (3.6) | (Chang et al., 2017) |
| Peer smoking | 1 (3.6) | (Bares, 2014) |
| Interleukin-6 levels | 1 (3.6) | (Perry et al., 2020) |
| Other | 3 (10.7) | (Duncan & Rees, 2005; Ranjit, Buchwald, et al., 2019; Wang et al., 2014) |

## **5. Meta-Analyses of Unadjusted Results**


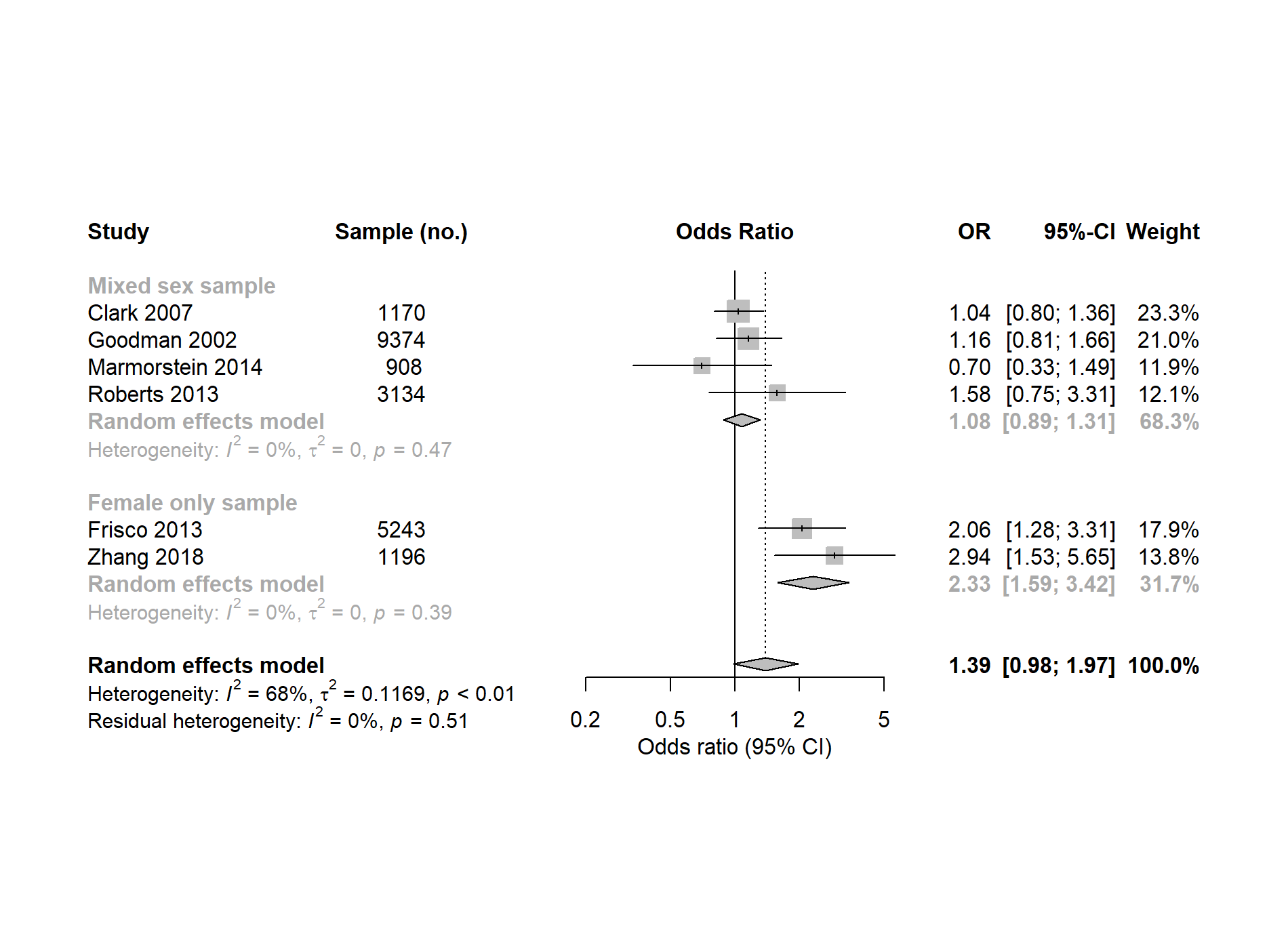
**S1 Fig. Meta-analysis of longitudinal association between high BMI at baseline and subsequent depression in young people.**

**S2 Fig. Meta-analysis of longitudinal association between smoking at baseline and subsequent depression in young people.**


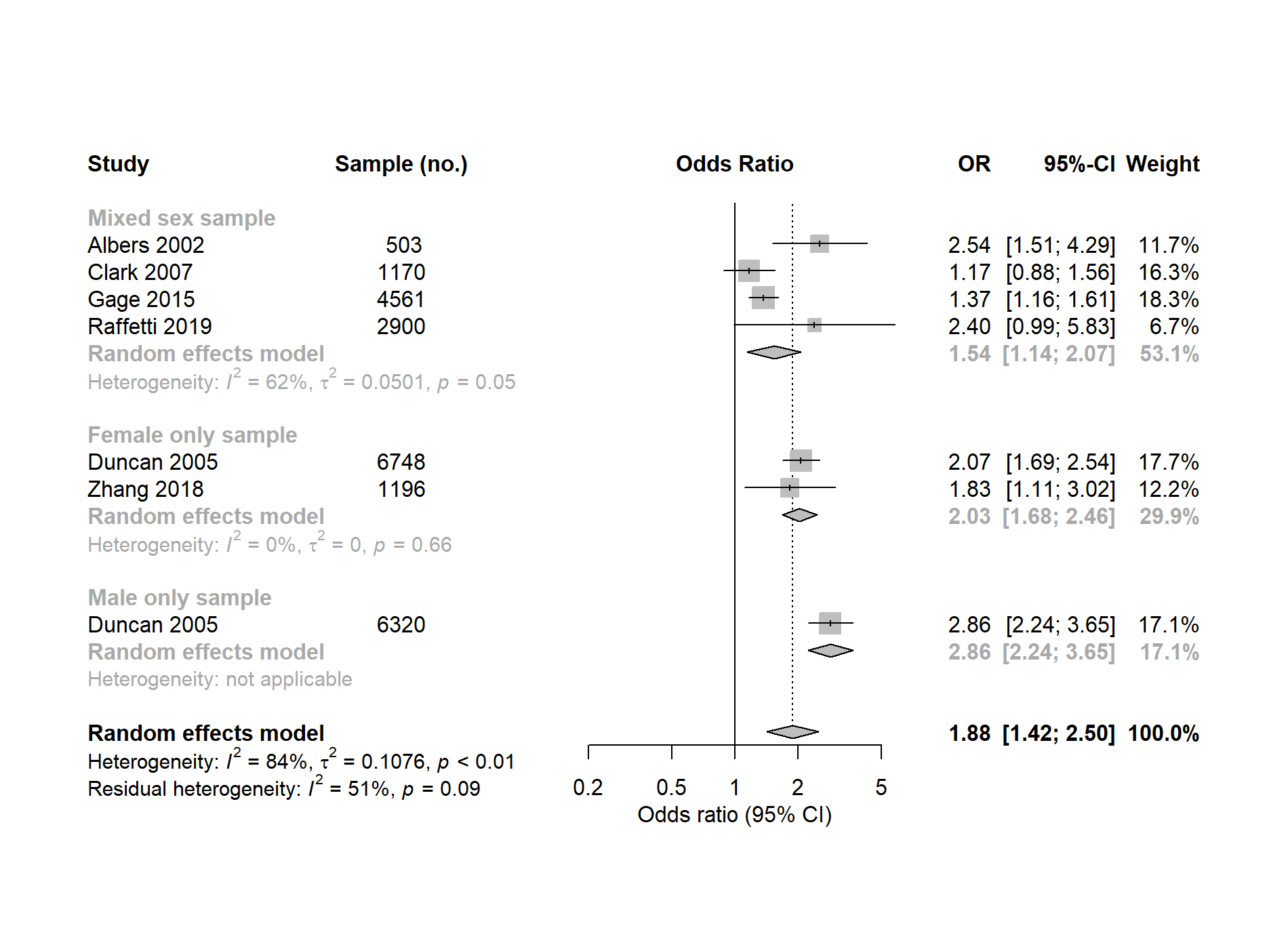


## **6. Meta-Analysis of Adjusted Results for Depressive Symptoms**

**
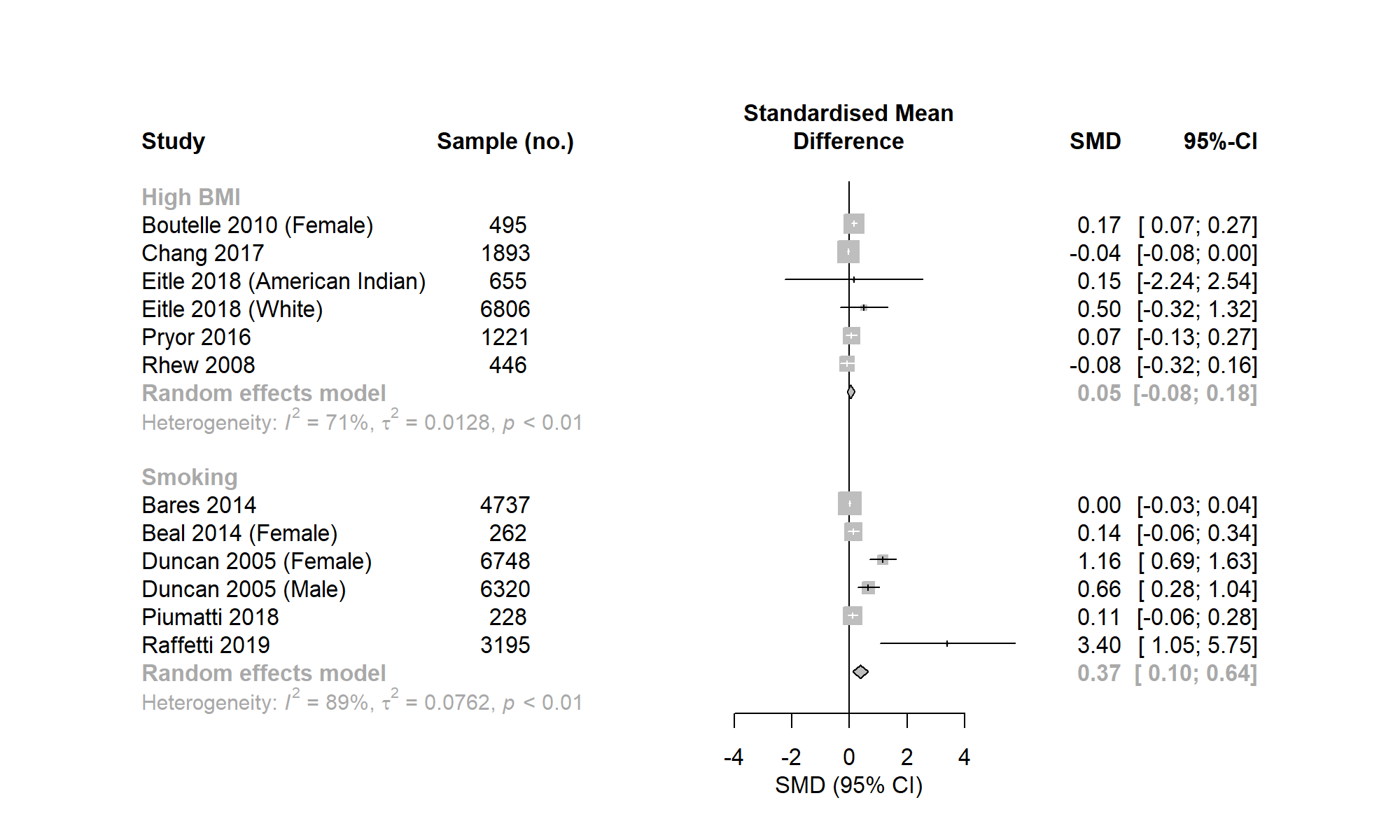
S3 Fig. Meta-analysis of longitudinal association between high BMI/smoking at baseline and subsequent depressive symptoms in young people.**

## **7. Sensitivity Analysis for Adjusted Results**

**S4 Fig. Meta-analysis, after poor quality studies removed, of longitudinal association between smoking at baseline and subsequent depression in young people.**


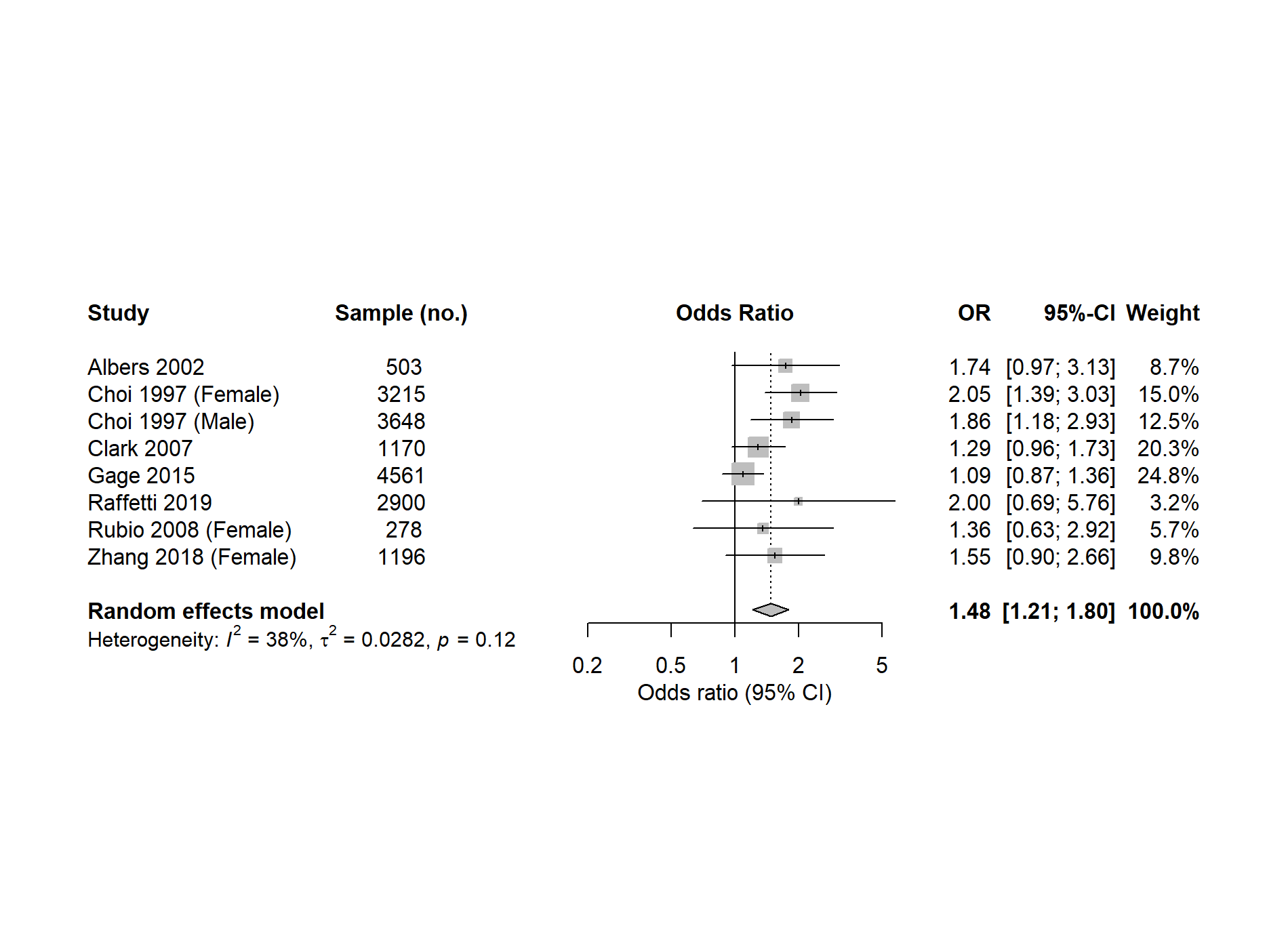


**S5 Fig. Meta-analysis, after female or male only studies removed, of longitudinal association between high BMI/smoking at baseline and subsequent depressive symptoms in young people.**


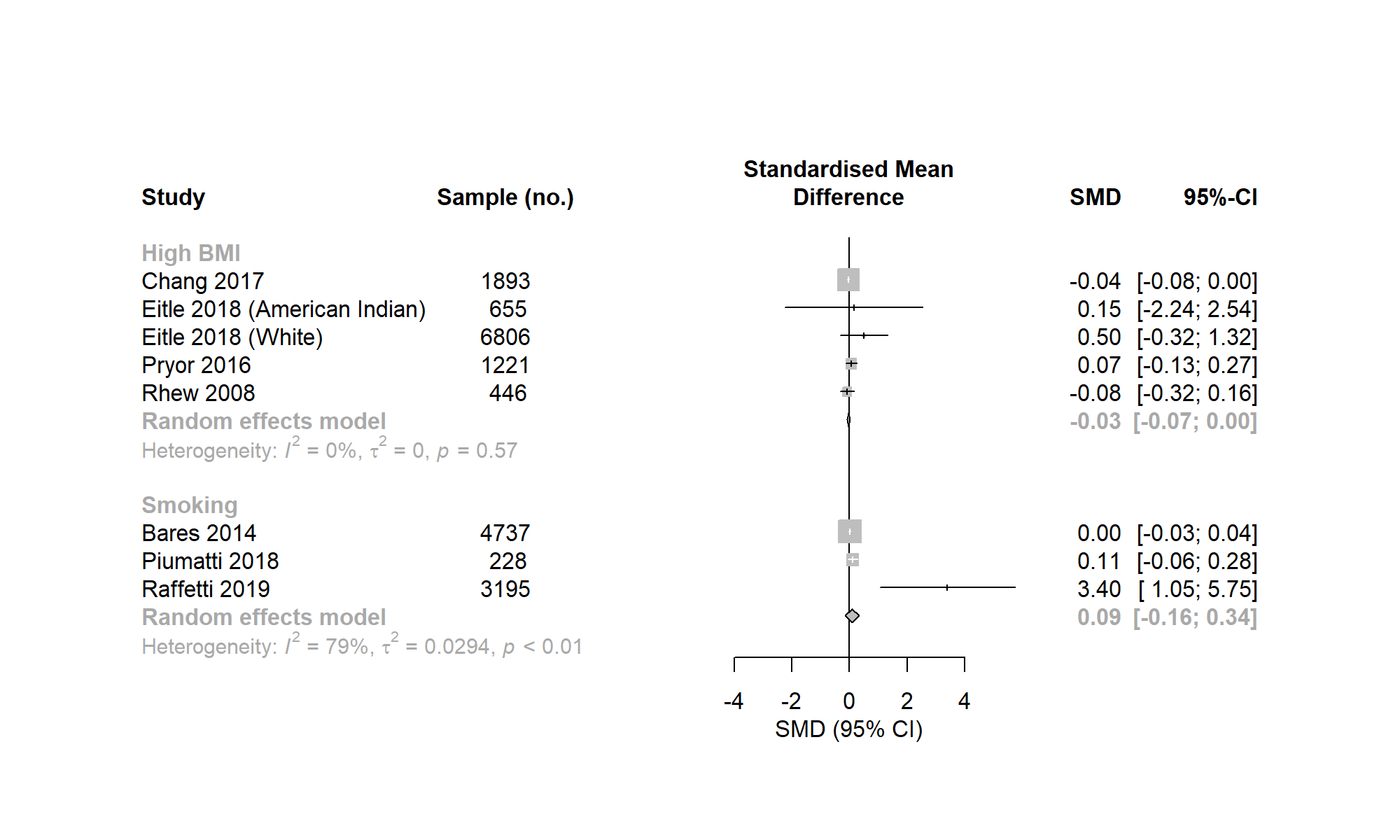


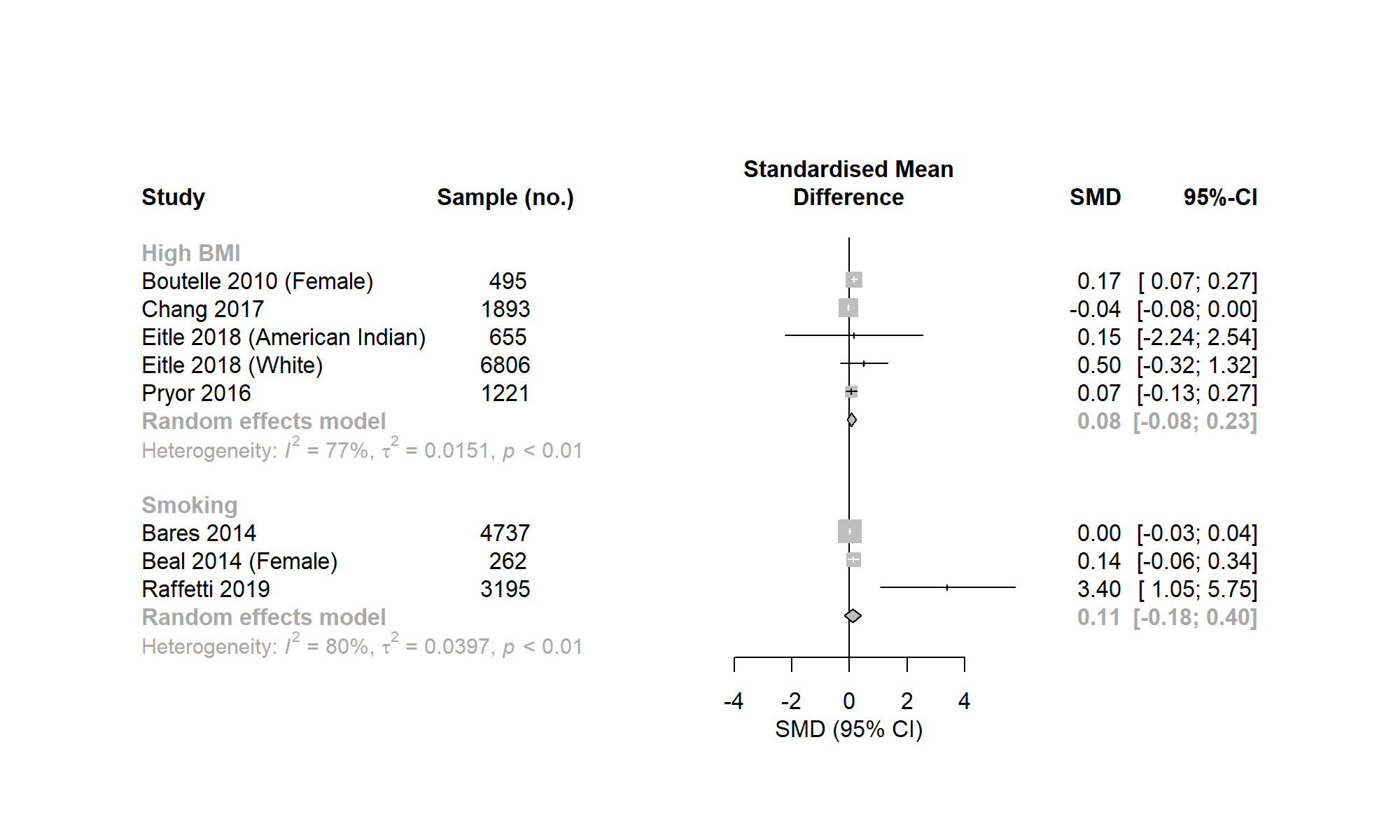
**S6 Fig. Meta-analysis, after poor quality studies removed, of longitudinal association between high BMI/smoking at baseline and subsequent depressive symptoms in young people.**

## **8. Publication Bias**

**S7 Fig.** **Adjusted odds ratios (standard error) of longitudinal association between high BMI at baseline and subsequent depression in young people.**

Egger’s test P-value = 0.17.


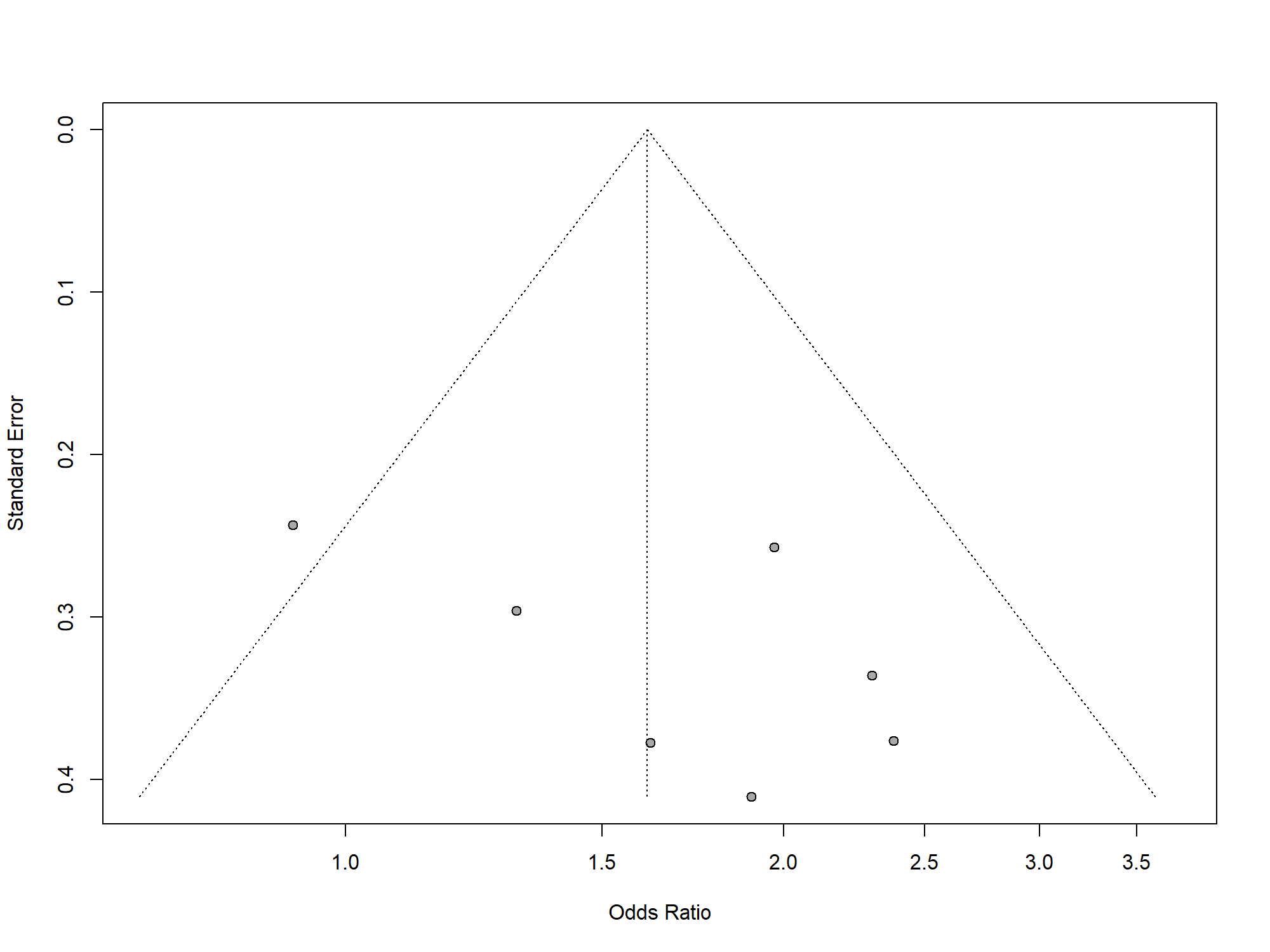


**S8 Fig.** **Adjusted odds ratios (standard error) of longitudinal association between smoking at baseline and subsequent depression in young people.**

Egger’s test P-value = 0.80.


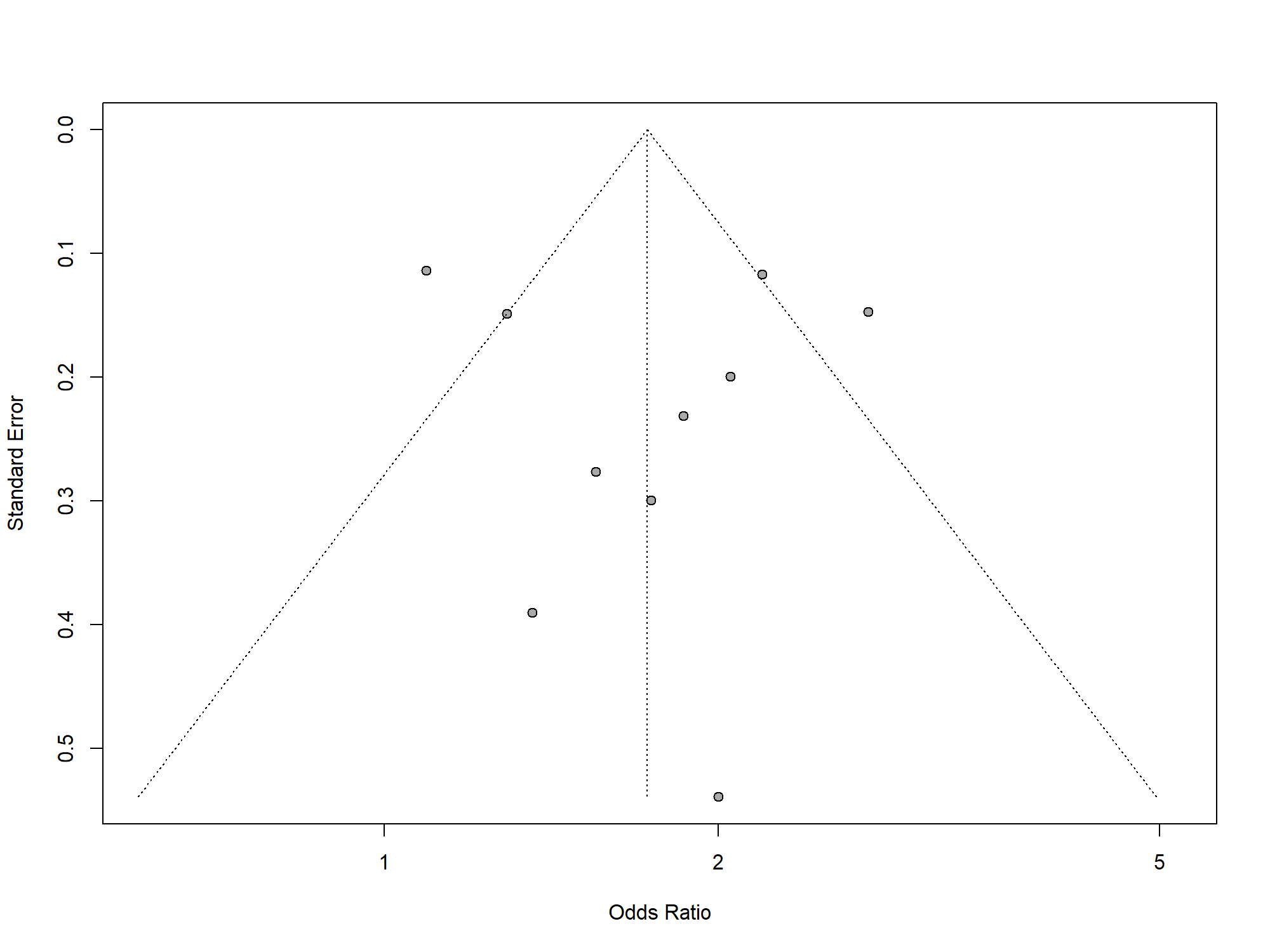


**S9 Fig.** **Adjusted standardised mean difference (standard error) of longitudinal association between high BMI at baseline and subsequent depressive symptoms in young people.**

Egger’s test P-value = 0.35.


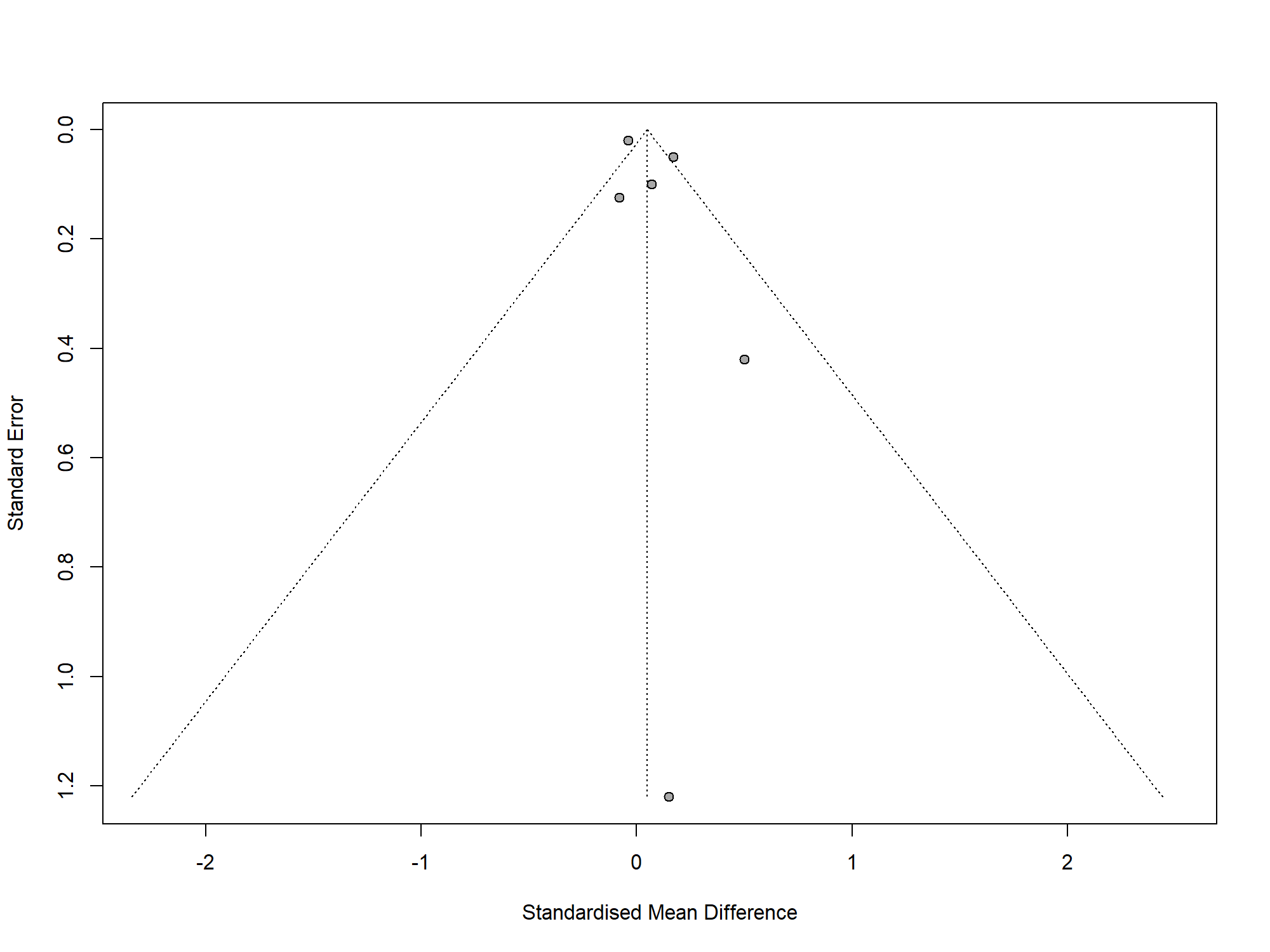


**S10 Fig. Adjusted standardised mean difference (standard error) of longitudinal association between smoking at baseline and subsequent depressive symptoms in young people.**


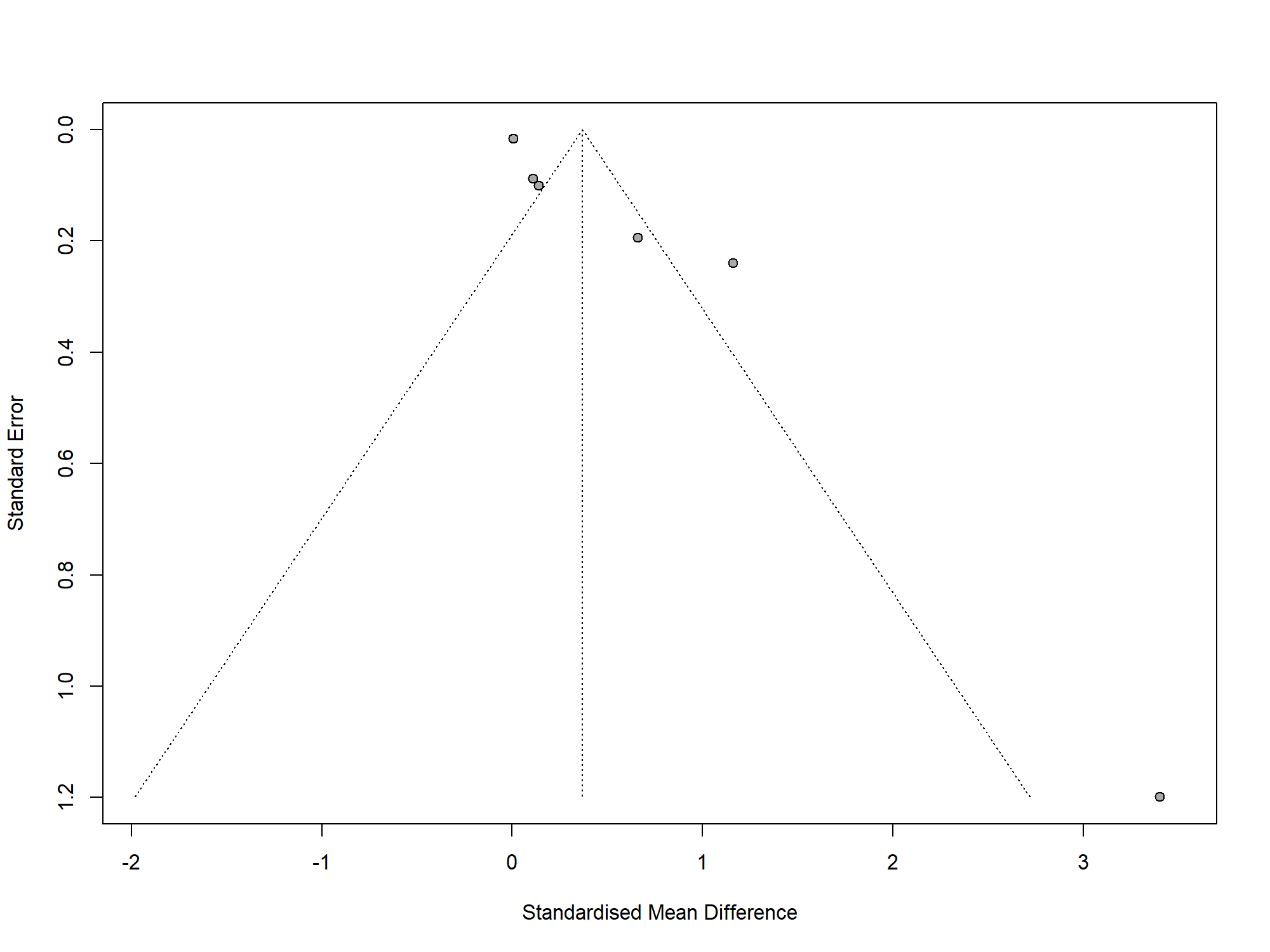


Egger’s test P-value = 0.01.

## **9. References**

Albers, A. B., & Biener, L. (2002). The Role of Smoking and Rebelliousness in the Development of Depressive Symptoms among a Cohort of Massachusetts Adolescents. *Preventive Medicine*, *34*(6), 625–631. https://doi.org/10.1006/pmed.2002.1029

Barefoot, J. C., & Schroll, M. (1996). Symptoms of Depression, Acute Myocardial Infarction, and Total Mortality in a Community Sample. *Circulation*, *93*(11), 1976–1980. https://doi.org/10.1161/01.CIR.93.11.1976

Bares, C. B. (2014). Gender, Depressive Symptoms and Daily Cigarette Use. *Journal of Dual Diagnosis*, *10*(4), 187–196. https://doi.org/10.1080/15504263.2014.961852

Beal, S. J., Negriff, S., Dorn, L. D., Pabst, S., & Schulenberg, J. (2014). Longitudinal Associations Between Smoking and Depressive Symptoms Among Adolescent Girls. *Prevention Science*, *15*(4), 506–515. https://doi.org/10.1007/s11121-013-0402-x

Berk, M., Williams, L. J., Jacka, F. N., O’Neil, A., Pasco, J. A., Moylan, S., … Maes, M. (2013). So depression is an inflammatory disease, but where does the inflammation come from? *BMC Medicine*, *11*(1), 200. https://doi.org/10.1186/1741-7015-11-200

Boutelle, K. N., Hannan, P., Fulkerson, J. A., Crow, S. J., & Stice, E. (2010). Obesity as a prospective predictor of depression in adolescent females. *Health Psychology*, *29*(3), 293–298. https://doi.org/10.1037/a0018645

Centers for Disease Control and Prevention (CDC). (2010). Prevalence of abnormal lipid levels among youths—United States, 1999-2006. *MMWR. Morbidity and Mortality Weekly Report*, *59*(2), 29–33.

Chaiton, M., Cohen, J. E., Rehm, J., Abdulle, M., & O’Loughlin, J. (2015). Confounders or intermediate variables? Testing mechanisms for the relationship between depression and smoking in a longitudinal cohort study. *Addictive Behaviors*, *42*, 154–161. https://doi.org/10.1016/j.addbeh.2014.11.026

Chaiton, M. O., Cohen, J. E., O’Loughlin, J., & Rehm, J. (2009). A systematic review of longitudinal studies on the association between depression and smoking in adolescents. *BMC Public Health*, *9*(1), 356. https://doi.org/10.1186/1471-2458-9-356

Chang, L.-Y., Chang, H.-Y., Wu, W.-C., Lin, L. N., Wu, C.-C., & Yen, L.-L. (2017). Body mass index and depressive symptoms in adolescents in Taiwan: Testing mediation effects of peer victimization and sleep problems. *International Journal of Obesity*, *41*(10), 1510–1517. https://doi.org/10.1038/ijo.2017.111

Choi, N. G., Kim, J., Marti, C. N., & Chen, G. J. (2014). Late-Life Depression and Cardiovascular Disease Burden: Examination of Reciprocal Relationship. *The American Journal of Geriatric Psychiatry*, *22*(12), 1522–1529. https://doi.org/10.1016/j.jagp.2014.04.004

Choi, W. S., Patten, C. A., Christian Gillin, J., Kaplan, R. M., & Pierce, J. P. (1997). Cigarette smoking predicts development of depressive symptoms among U.S. Adolescents. *Annals of Behavioral Medicine*, *19*(1), 42–50. https://doi.org/10.1007/BF02883426

Clark, C., Haines, M. M., Head, J., Klineberg, E., Arephin, M., Viner, R., … Stansfeld, S. A. (2007). Psychological symptoms and physical health and health behaviours in adolescents: A prospective 2-year study in East London. *Addiction*, *102*(1), 126–135. https://doi.org/10.1111/j.1360-0443.2006.01621.x

Duncan, B., & Rees, D. I. (2005). Effect of Smoking on Depressive Symptomatology: A Reexamination of Data from the National Longitudinal Study of Adolescent Health. *American Journal of Epidemiology*, *162*(5), 461–470. https://doi.org/10.1093/aje/kwi219

Eitle, D., & Eitle, T. M. (2018). Obesity, Overweightness, and Depressive Symptomology among American Indian Youth. *Journal of Racial and Ethnic Health Disparities*, *5*(6), 1305–1314. https://doi.org/10.1007/s40615-018-0479-9

Frisco, M. L., Houle, J. N., & Lippert, A. M. (2013). Weight Change and Depression Among US Young Women During the Transition to Adulthood. *American Journal of Epidemiology*, *178*(1), 22–30. https://doi.org/10.1093/aje/kws462

Gage, S. H., Hickman, M., Heron, J., Munafò, M. R., Lewis, G., Macleod, J., & Zammit, S. (2015). Associations of Cannabis and Cigarette Use with Depression and Anxiety at Age 18: Findings from the Avon Longitudinal Study of Parents and Children. *PLOS ONE*, *10*(4), e0122896. https://doi.org/10.1371/journal.pone.0122896

Gomes, A. P., Soares, A. L. G., Menezes, A. M. B., Assunção, M. C., Wehrmeister, F. C., Howe, L. D., & Gonçalves, H. (2019). Adiposity, depression and anxiety: Interrelationship and possible mediators. *Revista de Saúde Pública*, *53*, 103. https://doi.org/10.11606/S1518-8787.2019053001119

Goodman, E., & Capitman, J. (2000). Depressive symptoms and cigarette smoking among teens. *Pediatrics*, *106*(4), 748–755. https://doi.org/10.1542/peds.106.4.748

Goodman, E., & Whitaker, R. C. (2002). *A Prospective Study of the Role of Depression in the Development and Persistence of Adolescent Obesity*. 10.

Hammerton, G., Thapar, A., & Thapar, A. K. (2014). Association between obesity and depressive disorder in adolescents at high risk for depression. *International Journal of Obesity*, *38*(4), 513–519. https://doi.org/10.1038/ijo.2013.133

Hammerton, G., Harold, G., Thapar, A., & Thapar, A. (2013). Depression and blood pressure in high-risk children and adolescents: An investigation using two longitudinal cohorts. *BMJ Open*, *3*(9), e003206. https://doi.org/10.1136/bmjopen-2013-003206

Han, C., Liu, Y., Gong, X., Ye, X., & Zhou, J. (2019). Relationship between Secondhand Smoke Exposure and Depressive Symptoms: A Systematic Review and Dose–Response Meta-Analysis. *International Journal of Environmental Research and Public Health*, *16*(8), 1356. https://doi.org/10.3390/ijerph16081356

Hare, D. L., Toukhsati, S. R., Johansson, P., & Jaarsma, T. (2014). Depression and cardiovascular disease: A clinical review. *European Heart Journal*, *35*(21), 1365–1372. https://doi.org/10.1093/eurheartj/eht462

Hasin, D. S., Sarvet, A. L., Meyers, J. L., Saha, T. D., Ruan, W. J., Stohl, M., & Grant, B. F. (2018). Epidemiology of Adult DSM-5 Major Depressive Disorder and Its Specifiers in the United States. *JAMA Psychiatry*, *75*(4), 336. https://doi.org/10.1001/jamapsychiatry.2017.4602

Heredia, F. P. de, Gómez-Martínez, S., & Marcos, A. (2012). Obesity, inflammation and the immune system. *Proceedings of the Nutrition Society*, *71*(2), 332–338. https://doi.org/10.1017/S0029665112000092

Herrmann-Lingen, C., Meyer, T., Bosbach, A., Chavanon, M.-L., Hassoun, L., Edelmann, F., & Wachter, R. (2018). Cross-Sectional and Longitudinal Associations of Systolic Blood Pressure With Quality of Life and Depressive Mood in Older Adults With Cardiovascular Risk Factors: Results From the Observational DIAST-CHF Study. *Psychosomatic Medicine*, *80*(5), 468–474. https://doi.org/10.1097/PSY.0000000000000591

Hildrum, B., Mykletun, A., Stordal, E., Bjelland, I., Dahl, A. A., & Holmen, J. (2007). Association of low blood pressure with anxiety and depression: The Nord‐Trøndelag Health Study. *Journal of Epidemiology and Community Health*, *61*(1), 53–58. https://doi.org/10.1136/jech.2005.044966

Hiles, S. A., Baker, A. L., de Malmanche, T., McEvoy, M., Boyle, M., & Attia, J. (2015). The role of inflammatory markers in explaining the association between depression and cardiovascular hospitalisations. *Journal of Behavioral Medicine*, *38*(4), 609–619. https://doi.org/10.1007/s10865-015-9637-2

Hoare, E., Skouteris, H., Fuller-Tyszkiewicz, M., Millar, L., & Allender, S. (2014). Associations between obesogenic risk factors and depression among adolescents: A systematic review. *Obesity Reviews: An Official Journal of the International Association for the Study of Obesity*, *15*(1), 40–51. https://doi.org/10.1111/obr.12069

Holtzheimer, P. E., & Mayberg, H. S. (2011). Stuck in a Rut: Rethinking Depression and its Treatment. *Trends in Neurosciences*, *34*(1), 1–9. https://doi.org/10.1016/j.tins.2010.10.004

Huang, Y., Su, Y., Jiang, Y., & Zhu, M. (2020). Sex differences in the associations between blood pressure and anxiety and depression scores in a middle-aged and elderly population: The Irish Longitudinal Study on Ageing (TILDA). *Journal of Affective Disorders*, *274*, 118–125. https://doi.org/10.1016/j.jad.2020.05.133

Inouye, M., Abraham, G., Nelson, C. P., Wood, A. M., Sweeting, M. J., Dudbridge, F., … Samani, N. J. (2018). Genomic Risk Prediction of Coronary Artery Disease in 480,000 Adults. *Journal of the American College of Cardiology*, *72*(16), 1883–1893. https://doi.org/10.1016/j.jacc.2018.07.079

Juruena, M. F., Bocharova, M., Agustini, B., & Young, A. H. (2018). Atypical depression and non-atypical depression: Is HPA axis function a biomarker? A systematic review. *Journal of Affective Disorders*, *233*, 45–67. https://doi.org/10.1016/j.jad.2017.09.052

Kendler, K. S., Gardner, C. O., Fiske, A., & Gatz, M. (2009). Major depression and coronary artery disease in the Swedish twin registry: Phenotypic, genetic, and environmental sources of comorbidity. *Archives of General Psychiatry*, *66*(8), 857–863. https://doi.org/10.1001/archgenpsychiatry.2009.94

Kessler, R. C. (2012). The Costs of Depression. *The Psychiatric Clinics of North America*, *35*(1), 1–14. https://doi.org/10.1016/j.psc.2011.11.005

Kessler, R. C., McLaughlin, K. A., Green, J. G., Gruber, M. J., Sampson, N. A., Zaslavsky, A. M., … Williams, D. R. (2010). Childhood adversities and adult psychopathology in the WHO World Mental Health Surveys. *The British Journal of Psychiatry*, *197*(5), 378–385. https://doi.org/10.1192/bjp.bp.110.080499

Khandaker, G. M., Zuber, V., Rees, J. M. B., Carvalho, L., Mason, A. M., Foley, C. N., … Burgess, S. (2019). Shared mechanisms between coronary heart disease and depression: Findings from a large UK general population-based cohort. *Molecular Psychiatry*, 1. https://doi.org/10.1038/s41380-019-0395-3

Lippi, G., Montagnana, M., Favaloro, E. J., & Franchini, M. (2009). Mental depression and cardiovascular disease: A multifaceted, bidirectional association. *Seminars in Thrombosis and Hemostasis*, *35*(3), 325–336. https://doi.org/10.1055/s-0029-1222611

Luger, T. M., Suls, J., & Vander Weg, M. W. (2014). How robust is the association between smoking and depression in adults? A meta-analysis using linear mixed-effects models. *Addictive Behaviors*, *39*(10), 1418–1429. https://doi.org/10.1016/j.addbeh.2014.05.011

Luppino, F. S., Wit, L. M. de, Bouvy, P. F., Stijnen, T., Cuijpers, P., Penninx, B. W. J. H., & Zitman, F. G. (2010). Overweight, Obesity, and Depression: A Systematic Review and Meta-analysis of Longitudinal Studies. *Archives of General Psychiatry*, *67*(3), 220–229. https://doi.org/10.1001/archgenpsychiatry.2010.2

Mannan, M., Mamun, A., Doi, S., & Clavarino, A. (2016). Prospective Associations between Depression and Obesity for Adolescent Males and Females- A Systematic Review and Meta-Analysis of Longitudinal Studies. *PLoS ONE*, *11*(6). https://doi.org/10.1371/journal.pone.0157240

Marmorstein, N. R., Iacono, W. G., & Legrand, L. (2014). Obesity and depression in adolescence and beyond: Reciprocal risks. *International Journal of Obesity*, *38*(7), 906–911. https://doi.org/10.1038/ijo.2014.19

Monshouwer, K., Smit, F., Ruiter, M., Ormel, H., Verhulst, F., Vollebergh, W., & Oldehinkel, T. (2012). Identifying target groups for the prevention of depression in early adolescence: The TRAILS study. *Journal of Affective Disorders*, *138*(3), 287–294. https://doi.org/10.1016/j.jad.2012.01.026

Mühlig, Y., Antel, J., Föcker, M., & Hebebrand, J. (2016). Are bidirectional associations of obesity and depression already apparent in childhood and adolescence as based on high-quality studies? A systematic review. *Obesity Reviews*, *17*(3), 235–249. https://doi.org/10.1111/obr.12357

Munafò, M. R., Hitsman, B., Rende, R., Metcalfe, C., & Niaura, R. (2008). Effects of progression to cigarette smoking on depressed mood in adolescents: Evidence from the National Longitudinal Study of Adolescent Health. *Addiction*, *103*(1), 162–171. https://doi.org/10.1111/j.1360-0443.2007.02052.x

Niarchou, M., Zammit, S., & Lewis, G. (2015). The Avon Longitudinal Study of Parents and Children (ALSPAC) birth cohort as a resource for studying psychopathology in childhood and adolescence: A summary of findings for depression and psychosis. *Social Psychiatry and Psychiatric Epidemiology*, *50*(7), 1017–1027. https://doi.org/10.1007/s00127-015-1072-8

Osimo, E. F., Baxter, L. J., Lewis, G., Jones, P. B., & Khandaker, G. M. (2019). Prevalence of low-grade inflammation in depression: A systematic review and meta-analysis of CRP levels. *Psychological Medicine*, *49*(12), 1958–1970. https://doi.org/10.1017/S0033291719001454

Perry, B. I., Khandaker, G. M., Marwaha, S., Thompson, A., Zammit, S., Singh, S. P., & Upthegrove, R. (2020). Insulin resistance and obesity, and their association with depression in relatively young people: Findings from a large UK birth cohort. *Psychological Medicine*, *50*(4), 556–565. https://doi.org/10.1017/S0033291719000308

Piumatti, G. (2018). Motivation, health-related lifestyles and depression among university students: A longitudinal analysis. *Psychiatry Research*, *260*, 412–417. https://doi.org/10.1016/j.psychres.2017.12.009

Polanczyk, G. V., Salum, G. A., Sugaya, L. S., Caye, A., & Rohde, L. A. (2015). Annual research review: A meta-analysis of the worldwide prevalence of mental disorders in children and adolescents. *Journal of Child Psychology and Psychiatry, and Allied Disciplines*, *56*(3), 345–365. https://doi.org/10.1111/jcpp.12381

Pryor, L., Brendgen, M., Boivin, M., Dubois, L., Japel, C., Falissard, B., … Côté, S. M. (2016). Overweight during childhood and internalizing symptoms in early adolescence: The mediating role of peer victimization and the desire to be thinner. *Journal of Affective Disorders*, *202*, 203–209. https://doi.org/10.1016/j.jad.2016.05.022

Raffetti, E., Donato, F., Forsell, Y., & Galanti, M. R. (2019). Longitudinal association between tobacco use and the onset of depressive symptoms among Swedish adolescents: The Kupol cohort study. *European Child & Adolescent Psychiatry*, *28*(5), 695–704. https://doi.org/10.1007/s00787-018-1237-6

Ranjit, A., Buchwald, J., Latvala, A., Heikkilä, K., Tuulio-Henriksson, A., Rose, R. J., … Korhonen, T. (2019). Predictive Association of Smoking with Depressive Symptoms: A Longitudinal Study of Adolescent Twins. *Prevention Science*, *20*(7), 1021–1030. https://doi.org/10.1007/s11121-019-01020-6

Ranjit, A., Korhonen, T., Buchwald, J., Heikkilä, K., Tuulio-Henriksson, A., Rose, R. J., … Latvala, A. (2019). Testing the reciprocal association between smoking and depressive symptoms from adolescence to adulthood: A longitudinal twin study. *Drug and Alcohol Dependence*, *200*, 64–70. https://doi.org/10.1016/j.drugalcdep.2019.03.012

Rhew, I. C., Richardson, L. P., Lymp, J., McTiernan, A., McCauley, E., & Stoep, A. V. (2008). Measurement matters in the association between early adolescent depressive symptoms and body mass index. *General Hospital Psychiatry*, *30*(5), 458–466. https://doi.org/10.1016/j.genhosppsych.2008.06.008

Roberts, R. E., & Duong, H. T. (2013). Obese youths are not more likely to become depressed, but depressed youths are more likely to become obese. *Psychological Medicine*, *43*(10), 2143–2151. https://doi.org/10.1017/S0033291712002991

Roth, G. A., Abate, D., Abate, K. H., Abay, S. M., Abbafati, C., Abbasi, N., … Murray, C. J. L. (2018). Global, regional, and national age-sex-specific mortality for 282 causes of death in 195 countries and territories, 1980–2017: A systematic analysis for the Global Burden of Disease Study 2017. *The Lancet*, *392*(10159), 1736–1788. https://doi.org/10.1016/S0140-6736(18)32203-7

Rubio, D. M., Kraemer, K. L., Farrell, M. H., & Day, N. L. (2008). Factors Associated With Alcohol Use, Depression, and Their Co-occurrence During Pregnancy. *Alcoholism: Clinical and Experimental Research*, *32*(9), 1543–1551. https://doi.org/10.1111/j.1530-0277.2008.00705.x

Shin, J. Y., Suls, J., & Martin, R. (2008). Are Cholesterol and Depression Inversely Related? A Meta-analysis of the Association Between Two Cardiac Risk Factors. *Annals of Behavioral Medicine*, *36*(1), 33–43. https://doi.org/10.1007/s12160-008-9045-8

Speed, M. S., Jefsen, O. H., Børglum, A. D., Speed, D., & Østergaard, S. D. (2019). Investigating the association between body fat and depression via Mendelian randomization. *Translational Psychiatry*, *9*(1), 1–9. https://doi.org/10.1038/s41398-019-0516-4

Stang, A. (2010). Critical evaluation of the Newcastle-Ottawa scale for the assessment of the quality of nonrandomized studies in meta-analyses. *European Journal of Epidemiology*, *25*(9), 603–605.

Sutaria, S., Devakumar, D., Yasuda, S. S., Das, S., & Saxena, S. (2019). Is obesity associated with depression in children? Systematic review and meta-analysis. *Archives of Disease in Childhood*, *104*(1), 64–74. https://doi.org/10.1136/archdischild-2017-314608

Van der Kooy, K., van Hout, H., Marwijk, H., Marten, H., Stehouwer, C., & Beekman, A. (2007). Depression and the risk for cardiovascular diseases: Systematic review and meta analysis. *International Journal of Geriatric Psychiatry*, *22*(7), 613–626. https://doi.org/10.1002/gps.1723

Wang, H., Leung, G. M., & Schooling, C. M. (2014). Life Course Adiposity and Adolescent Depressive Symptoms Among Hong Kong Adolescents. *Journal of Adolescent Health*, *55*(3), 408–414. https://doi.org/10.1016/j.jadohealth.2014.03.009

Wei, Y.-G., Cai, D.-B., Liu, J., Liu, R.-X., Wang, S.-B., Tang, Y.-Q., … Wang, F. (2020). Cholesterol and triglyceride levels in first-episode patients with major depressive disorder: A meta-analysis of case-control studies. *Journal of Affective Disorders*, *266*, 465–472. https://doi.org/10.1016/j.jad.2020.01.114

WHO Study Group of Young People. (1986). *Young people’s health - a challenge for society: Report of a WHO Study Group on Young People and “Health for All by the Year 2000.”* World Health Organization. Retrieved from https://apps.who.int/iris/handle/10665/41720

Willerson, J. T., & Ridker, P. M. (2004). Inflammation as a Cardiovascular Risk Factor. *Circulation*, *109*(21), 2–10. https://doi.org/10.1161/01.CIR.0000129535.04194.38

Wilson, P. W., D’Agostino, R. B., Levy, D., Belanger, A. M., Silbershatz, H., & Kannel, W. B. (1998). Prediction of coronary heart disease using risk factor categories. *Circulation*, *97*(18), 1837–1847. https://doi.org/10.1161/01.cir.97.18.1837

Wilson, P. W. F., Castelli, W. P., & Kannel, W. B. (1987). Coronary risk prediction in adults (The Framingham Heart Study). *The American Journal of Cardiology*, *59*(14), G91–G94. https://doi.org/10.1016/0002-9149(87)90165-2

Zhang, X. C., Woud, M. L., Becker, E. S., & Margraf, J. (2018). Do health-related factors predict major depression? A longitudinal epidemiologic study. *Clinical Psychology & Psychotherapy*, *25*(3), 378–387. https://doi.org/10.1002/cpp.2171
